# A search engine to identify pathway genes from expression data on multiple organisms

**DOI:** 10.1186/1752-0509-1-20

**Published:** 2007-05-04

**Authors:** Chunnuan Chen, Matthew T Weirauch, Corey C Powell, Alexander C Zambon, Joshua M Stuart

**Affiliations:** 1 Department of Biomolecular Engineering, University of California, Santa Cruz, California, 95064, USA; 2 Department of Medicine, Gladstone Institute of Cardiovascular Disease, San Francisco, California 94158, USA

## Abstract

**Background:**

The completion of several genome projects showed that most genes have not yet been characterized, especially in multicellular organisms. Although most genes have unknown functions, a large collection of data is available describing their transcriptional activities under many different experimental conditions. In many cases, the coregulatation of a set of genes across a set of conditions can be used to infer roles for genes of unknown function.

**Results:**

We developed a search engine, the Multiple-Species Gene Recommender (MSGR), which scans gene expression datasets from multiple organisms to identify genes that participate in a genetic pathway. The MSGR takes a query consisting of a list of genes that function together in a genetic pathway from one of six organisms: *Homo sapiens*, *Drosophila melanogaster*, *Caenorhabditis elegans*, *Saccharomyces cerevisiae*, *Arabidopsis thaliana*, and *Helicobacter pylori*. Using a probabilistic method to merge searches, the MSGR identifies genes that are significantly coregulated with the query genes in one or more of those organisms. The MSGR achieves its highest accuracy for many human pathways when searches are combined across species. We describe specific examples in which new genes were identified to be involved in a neuromuscular signaling pathway and a cell-adhesion pathway.

**Conclusion:**

The search engine can scan large collections of gene expression data for new genes that are significantly coregulated with a pathway of interest. By integrating searches across organisms, the MSGR can identify pathway members whose coregulation is either ancient or newly evolved.

## Background

One of the current challenges of genetics is to identify the genes involved in the major physical, regulatory, and signaling events that direct molecular processes. Even for well-characterized genetic pathways, our knowledge about the genes involved is often incomplete. However, new high-throughput datasets make it possible to search for hitherto unclassified genes that display coordinated activity with known members of a pathway.

Genes that participate in the same pathway or whose products are part of the same protein complex are often coregulated. For example, genes encoding proteins that bind each other in a complex may be coregulated to maintain its stoichiometry. Similarly, gene products that signal to each other in a phosphorylation cascade must be present together to communicate. Thousands of DNA microarray experiments have been conducted to investigate various cellular processes [1]. Evidence for functional relatedness may be found by searching this growing body of gene expression data [2,3].

The clustering of genes according to shared expression patterns across diverse microarray experiments has provided insights into gene regulation and has assigned function to previously uncharacterized genes. Clustering methods are successful when the set of conditions is small [2,4,5], but are limited when the set of conditions is large [6,7]. As gene expression repositories grow, finding the experimental context in which a pathway is coregulated will be a key step in identifying previously unknown genes in a pathway. Biclustering methods such as Plaid [8] and coupled-two way clustering [4,9-11] identify subsets of experiments and genes simultaneously. However, these methods are unsupervised and do not use existing information about a pathway to guide the search for new members. Methods that incorporate such information are needed when a gene may belong to multiple pathways or when a pathway is activated under a small set of experimental conditions.

Integrating expression data across organisms is another promising approach to increase the accuracy of pathway discovery. Coexpression identified in a single organism may result from inherent biological or experimental noise. If identified in multiple organisms, however, coexpression is more likely to reflect a functional link between genes because the coordinated regulation occurs in completely independent datasets and has survived millions of years of selection.

Our goal is to develop a search engine that uses gene expression data to predict new pathway members with high accuracy. Given a set of genes as input, called the *query*, the search engine outputs a *search ordering *in which all of the genes in the genome are ranked according to their coregulation with the query genes. If the pathway genes are loosely coregulated, or the pathway is coregulated with many other pathways in parallel, the pathway genes may not sort to the top of the search ordering. This complicates the identification of new members because many unrelated genes will instead sort toward the top.

**Figure 1:**
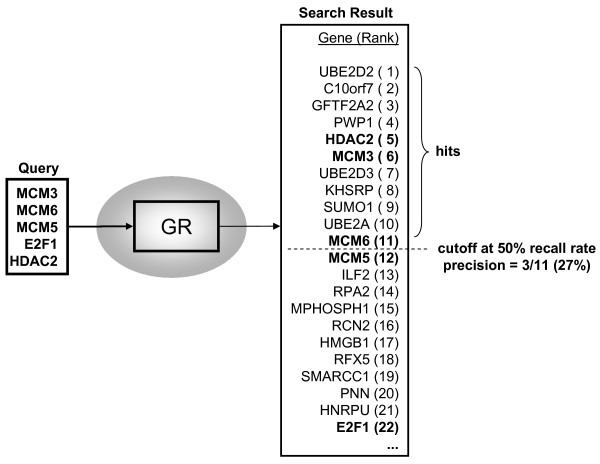
**The GeneRecommender search engine**. A query set of five genes that participate in the cell cycle is given to the GeneRecommender search engine. The search engine outputs a search ordering, which is an ordered list of genes sorted by the extent of their coregulation with the query genes. A hit list is defined as the set of top-scoring genes that include half of the original query genes.

We hypothesized that integrating search results obtained on different organisms would improve our ability to identify pathway members. If a pathway's genes are loosely coregulated in not one, but several organisms, the pathway members and their orthologs may consistently rank higher than unrelated genes in searches across the organisms. A search engine that makes use of this tendency may produce a more accurate result. To test this hypothesis, we developed a search engine, the Multiple-Species Gene Recommender (MSGR), which combines search results obtained by the GeneRecommender [7] run on multiple organisms. The GeneRecommender is a search engine that identifies genes highly coregulated with the query genes using data from a single organism. Each microarray hybridization, which contains gene expression measurements for a major fraction of genes in the genome, is scored by the GeneRecommender to reflect the extent to which the expression levels of the query genes are coregulated with each other. High scores indicate significantly coordinated up- or down-regulation. The hybridizations with the highest scores are chosen for the next step. The GeneRecommender then returns a search ordering in which the genes are ranked by their coregulation with the query genes under the chosen hybridizations (see Figure 1). Genes at the top of the search ordering are considered to have higher coregulation with the query genes. Here we show that combining search results across species using the MSGR improves our ability to predict gene function for several pathways.

## Results

### Searching single organisms

To identify pathways for which combining multiple searches across organisms could improve search accuracy, we assessed the ability of the GeneRecommender to predict pathway membership using data from only a single organism. We extended the GeneRecommender search engine to scan six organisms, including *C. elegans*, for which the GeneRecommender was designed. Gene expression data were assembled from 5962 *Homo sapiens *(human), 334 *Drosophila melanogaster *(fly), 1209 *Caenorhabditis elegans *(worm), 753 *S. cerevisiae *(yeast), 649 *Arabidopsis thaliana *(plant), and 293 *Helicobacter pylori *(bacterium) microarray hybridizations. A diverse range of experimental conditions was included for each of these distantly related organisms (see Methods). We tested the GeneRecommender on 51 pathways from the GenMAPP pathway collection [12], a diverse and approximately nonredundant collection of human pathways derived from Gene Ontology [13] and KEGG [14], and pathways contributed by experts in the field (see Supplemental Table 1).

To determine the accuracy of the GeneRecommender when searching a single organism, we measured the precision of the search orderings returned for each of the six organisms. Precision was computed as the fraction of query genes in a *hit list*, formed by truncating the search ordering at a cutoff that included half of the original query genes, guaranteeing a 50% recall rate for the hit list. Half, but not all, of the query genes were included in the hit list, rendering the estimated precision insensitive to genes erroneously included in the query list or to errors in the microarray data (e.g., poor-quality probes for one or a few of the query genes). In the example shown in Figure 1, the hit list contains 11 genes because three of the five human *Cell Cycle *query genes were ranked in the top 11 of the 22 genes in the search ordering. The precision for the hit list shown in this example is therefore 3/11 (0.27).

A search result was considered to be "highly precise" if it attained precision levels of at least 20%. At this level of precision, five genes on average would need to be tested to ensure that a related member of the pathway was tested. A search result was considered to be "moderately precise" if it attained precision levels above 2%. At this level, 50 genes would need to be tested to ensure that a related member was tested.

To obtain a conservative estimate of the precision, we used query ranks obtained from cross-validation rather than a single search in which all of the query genes were included. We performed a cross-validation in which 80% of the pathway genes were used as the input query to obtain a search ordering from which the ranks of the remaining 20% were recorded. By iteratively withholding a distinct 20% of the query genes, we collected cross-validation ranks for each of the query genes (see Methods).

For each GenMAPP pathway, we constructed a corresponding GenMAPP pathway by replacing each human gene from the original pathway with its putative ortholog in the target species (see Methods). Of the 51 GenMAPP pathways, 39 had the minimum number of five genes, required for use with the GeneRecommender, in at least two organisms. A *coexpression score *for each pathway was computed as the sum of a pathway's precisions in each of the six organisms. High coexpression scores indicated pathways in which the GeneRecommender identified either a highly precise hit list in one organism or moderately precise hit lists in several organisms.

**Figure 2:**
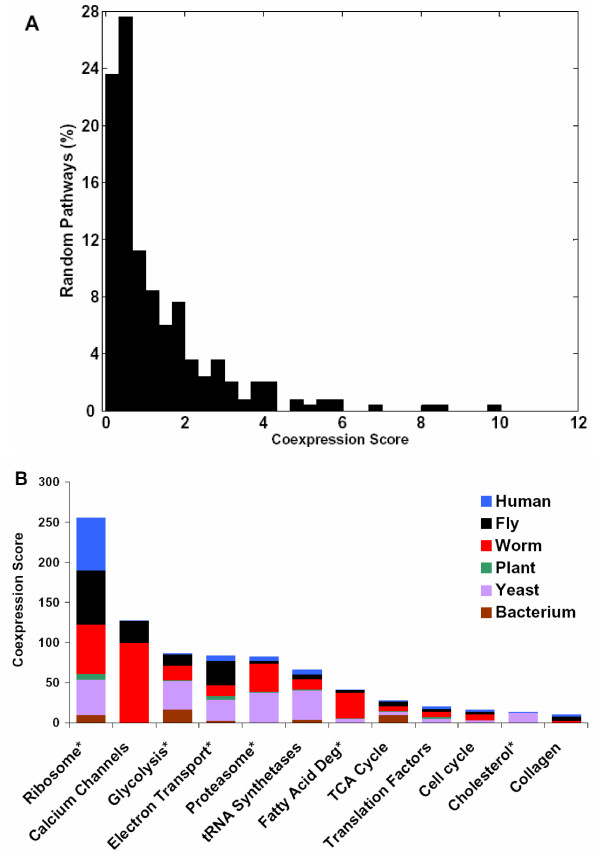
**Performance of the search engine using data from separate organisms**. **A. **Random control pathways were scored by the GeneRecommender in all six organisms, and the coexpression score was computed from the search orderings as the sum of the precision at the 50% recall rate across all six organisms. **B. **Precision scores of the top-scoring pathways from the GenMAPP database. Each bar corresponds to a single pathway. Colored segments indicate the precision at the 50% recall rate for each organism. The height of each bar corresponds to the pathway's coexpression score. Asterisks indicate names that were modified to fit on the plot: *Ribosome*, *Cytoplasmic Ribosomal Proteins*; *Glycolysis*, *Glycolysis and Gluconeogenesis*; *Electron Transport*, *Electron Transport Chain*; *Proteasome*, *Proteasome Degradation*; *Fatty Acid Deg*, *Fatty Acid Degradation*; *Cholesterol*, *Cholesterol Biosynthesis*.

To identify pathways with significant coexpression scores, we ran the GeneRecommender on 1000 randomly generated queries of the same size as the input queries (see Figure 2A). The mean of the coexpression scores for the random queries was 1.31 ± 1.49 (SD). A coexpression score of at least 10 was significant at the 0.001 level since only a single random query obtained a better precision (10.03, see Figure 2A).

Twelve of the 39 GenMAPP pathways tested had significant coexpression scores (see Figure 2B). Six of these pathways had already been shown to be tightly coregulated in gene expression databases: *Cytoplasmic Ribosomal Proteins*, *Glycolysis and Gluconeogenesis*, *Electron Transport Chain*, *Proteasomal Degradation*, *tRNA Synthetases*, and *Fatty Acid Degradation *[2]. These pathways contain genes involved in basic cellular processes (e.g., cell growth and energy generation). Pathways that did not score well were involved in specific biosynthetic processes (e.g., *Steroid Biosynthesis*, *Eicosanoid Synthesis*, and *Methionine Metabolism*), G-protein coupled receptor pathways, *Apoptosis*, *Ovarian Infertility Genes*, and *Blood Clotting Cascade*. The scores may have been low because the current gene expression databases lacked conditions under which the genes in these pathways are coordinately regulated or because these pathways may represent biological processes with high levels of evolutionary divergence.

To assess the ability of the MSGR to identify genes involved in pathways specific to animals, we tested it on two high-scoring pathways, *Calcium Channels *and *Collagens*. The *Calcium Channels *pathway comprises a multifunctional set of genes that regulate intracellular calcium levels (Supplemental Table 2) and plays a role in hormone and neurotransmitter release, muscle contraction, and immune cell activation. The GeneRecommender achieved a high coexpression score for the *Calcium Channels *category (127) due to its high-precision searches in both fly (27%) and worm (100%). We therefore chose the *Calcium Channels *pathway as a positive control. If a high proportion of the genes that rank to the top of the fly result also rank to the top of the worm result, then the multiple-species approach should perform well on this pathway.

The *Collagens *pathway represents a more challenging test of the MSGR's ability than the *Calcium Channels *pathway. The GenMAPP *Collagens *pathway contains 49 human genes, including the collagen structural genes and other cell-adhesion genes with important roles in the formation of basement membranes, cytoskeletal structures, and the extracellular matrix (Supplemental Table 2) [15]. The single-species results for the *Collagens *pathway were moderately precise (5.1% in fly, 2.7% in worm, and 2.4% in human). The pathway's coexpression score of 10.2 was slightly better than that seen in the random controls (Figure 2B). Thus, for the multiple-species approach to succeed for the *Collagens *pathway, it must combine multiple weak signals into a more predictive result

### Combining search orderings across multiple organisms to investigate multicellular pathways

**Figure 3:**
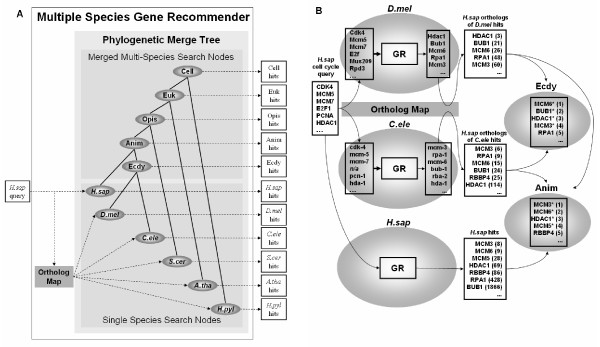
**Overview of the MSGR**. **A. **The genes mapped through the BTP table, denoted "Ortholog Map," are supplied as queries to an organism-specific GeneRecommender [7] search (gray ovals). The search orderings of each organism (dashed arrows) and of all ancestral search nodes that are parents in the phylogenetic merge tree (solid arrows) are reported. The five ancestral merge nodes are Ecdysozoan (Ecdy), Animal (Anim), Opisthokont (Opis), Eukaryote (Euk), and Cellular (Cell). *H.sap, Homo sapiens; D.mel, Drosophila melanogaster*; *C.ele,Caenorhabditis elegans*;*S.cer, Saccharomyces cerevisiae*; *A.tha, Arabidopsis thaliana*; *H.pyl*, *Helicobacter pylori*. **B. **Illustration of the merging of search orderings using a human cell-cycle pathway as the query. Nodes in the phylogenetic merge tree are shown as gray ovals. GR, GeneRecommender. Boxes represent search orderings output as lists of genes. Numbers in parentheses are the ranks of the genes in the search ordering.

Next, we asked whether the search orderings obtained in separate organisms for the *Calcium Channels *and *Collagens *pathways could be combined to complement one another and produce a more accurate search ordering. The MSGR generates new search orderings by combining two or more of the GeneRecommender's search orderings, using a data structure called a *phylogenetic merge tree *(PMT) to determine which species-specific search orderings to combine (see Figure 3A and Methods). The MSGR stores the search ordering for a single organism at an *organism node *corresponding to the organism's phylogenetic position in the tree. The MSGR computes a search ordering for a common ancestor by combining the search orderings stored in all of the organism nodes beneath the *ancestral node *in the tree. The MSGR only produces combinations present in the PMT to allow the search engine to scale to a large number of organisms while still producing evolutionarily coherent merges. Nodes in the tree are labeled with an initial uppercase letter to distinguish its use from an organism or ancestor (e.g., "Human" for search orderings collected using only human data and "Animal" for search orderings obtained by merging results from the multicellular animals).

Each node in the PMT contains a different ranking of genes. Figure 3B shows an example of how the MSGR combines human, fly, and worm search orderings for a human cell cycle query at the Animal PMT node. At such an ancestral node in the tree, genes are ranked according to a joint *P *value that reflects the degree to which a gene sorted to the top of the search orderings in each of the organisms beneath that node (see Methods). More significant (smaller) *P *values correspond to genes that are ranked toward the top of multiple search orderings. For example, in Figure 3B, *HDAC1 *sorts higher than *MCM3 *in the Ecdysozoan search ordering because the probability of achieving a rank of 3 in fly and a rank of 114 in worm is lower than the probability of achieving a rank of 60 in fly and 6 in worm. Rather than simply summing up or multiplying the ranks across different searches, the MSGR computes a *P *value that factors in the number of genes considered in each species and the statistical chance of achieving the observed ranks independently at random (see Methods and the Appendix in Additional file 1). Results from fly and worm are combined at the Ecdysozoa node of the tree, yielding a search ordering specific to those two molting organisms. Results from human, fly, and worm are combined at the Animal node in the tree, yielding a search ordering specific to those three multicellular organisms. Some combinations of organisms may yield more accurate results than others for a particular pathway. For this reason, the search engine inspects the precision of the search orderings at every ancestral and organism node in the PMT to identify good combinations.

To translate a query from a source organism to a target organism, the MSGR uses only genes conserved between the two species. For a gene in the source organism's query, the MSGR adds a putative ortholog to the target organism's query. To perform this mapping, we use a single best-matching protein, which we refer to as the best target protein (BTP), rather than a collection of related proteins, to avoid including large gene families containing paralogs of possibly diverged function (see Methods). For the remainder of the discussion, we assume that human is the source organism; however, genes from any one of the included organisms can be used to initiate a search.

### Multiple-species search for genes involved in calcium signaling

The MSGR obtained high precision for the *Calcium Channels *query when it combined the search orderings from all three multicellular organisms at the Animal PMT node. The human query set supplied to the MSGR had 26 genes. Predicted orthologs in either worm or fly were found for seven of these genes (see Additional file 7, Supplemental Table 2). The remaining 19 genes did not have best-target proteins in worm or fly either because they belonged to duplicated gene groups (12 genes) or because no matching gene could be found in either organism (seven genes). After combining human, worm, and fly search orderings in the Animal node, the MSGR identified a hit list with a precision at the 50% recall rate of 50% (4 of 8 query genes lie above dashed line in Table 1). Of the seven original genes in the pathway, four scored in the top 10 of the Animal node results (with ranks 1, 2, 3, and 8), and three received poorer ranks (359, 531, and 861).

Inspecting the known functions of the genes returned by the MSGR suggested that the search engine identified genes coregulated with a conserved calcium-dependant signaling pathway involved in neuromuscular function. Of the top 25 hits for *Calcium Channels*', 21 genes were not included in the original query. Out of these, 16 had functions related to neuromuscular processes (see Table 1), including six that participate in neural or muscular signaling, three that are involved in muscle contraction, and two that are expressed in neurons but with unknown roles. Several genes had no known association with neuromuscular signaling, but were known to be involved in other signaling functions. Interestingly, of the three ryanodine receptors, only the neuron-specific ryanodine receptor 2 (RYR2) [16] was returned; the two non-neural receptors were excluded from the list. The functions of the known genes are consistent with a core calcium-dependent signaling pathway with a specific role in neuromuscular function (for a list of the 500 top-scoring genes see Additional file 8, Supplemental Table 3).

Two of the genes in the list, SHOC2 and USP11, had no prior association with calcium-dependent signaling. SHOC2 encodes an ortholog of the *C. elegans *soc-2 gene, which is expressed in neurons and muscle of adult worms. USP11 is a ubiquitin-specific protease that removes ubiquitin groups from specific proteins to provide "switch-like" behavior in response to multiple different intracellular events, including signal transduction [17]. Recent studies in *D. melanogaster *also link ubiquitin modification with a signal transduction cascade initiated after immune cell activation [18].

To test the hypothesis that the genes returned by the MSGR participate in a common signaling pathway implicated in neural function, we looked for putative *cis*-regulatory control sequences (see Methods). Seven transcription factor binding sites were overrepresented in the upstream regions of 24 of 25 genes from this group (see Additional file 10, Supplemental Table 5). Among the transcription factors with binding sites represented in the MSGR's search ordering were the neuronal factors ZIC1 and ZIC2. The ZIC family is a group of zinc finger proteins that are expressed in the cerebellum and regulate several brain-specific genes [19]. Of the top 25 hits, 13 contained a ZIC1 binding site and eight contained a ZIC2 binding site (see Additional file 11, Supplemental Table 6). From simulations with collections of 25 genes drawn randomly from the genome, we expected 6.9 (*P *< 0.004) genes with hits for ZIC1 and 2.8 (*P *< 0.004) genes with hits for ZIC2. Significant hits were also found for the MYC-associated zinc finger (MAZ) protein, which interacts with the neural survival factor netrin-1 [20]. The upstream sequences of the genes identified in the *Calcium Channels *hit list were also enriched for binding sites for the *BRAIN2 *transcription factor. *BRAIN2 *is required for establishing mammalian neural cell lineages [21] and is expressed in various parts of the brain [22,23].

More *Calcium Channels *genes than expected by chance had upstream sequences containing binding sites related to immune system functions. For example, enrichment was found for the binding site of the myeloid differentiation factor MZF1, a transcription factor involved in the differentiation of immune responsive myeloid cells from hematopoietic stem cell progenitors. Calcium signaling plays a fundamental role in immune cell signaling and activation [24]. For example, T-cell activation triggers calcium release from vacuolar stores [25]. This suggests the MSGR identified a set of genes regulated by both neural- and immune-related factors. These genes may represent a core calcium-dependent signaling pathway that functions in multiple contexts.

Next, we analyzed the upstream sequences of the *Calcium Channels *query genes that were excluded from the Animal hit list based on their expression patterns. Of the three excluded query genes, only one had a hit to any of the seven binding sites enriched for the *Calcium Channels *hit list (ITPR3 had hits to MAZ and MZF1). This finding demonstrates the MSGR's ability to divide the query genes into a set of genes that are strongly coexpressed with each other and a set of genes that are not coexpressed with each other, as supported by the independent transcription factor binding site analysis.

### Multiple-species search for genes involved in cell adhesion

To determine if the MSGR could combine the moderately precise search orderings from human, fly, and worm to more accurately predict new members, we used the MSGR to scan for new genes with functions related to the *Collagens *pathway. Twelve genes from the human *Collagens *query set had putative orthologs in worm (nine) and/or fly (10) (see Additional file 7, Supplemental Table 2).

The query genes sorted significantly toward the top of the multiple-species search orderings. For example, at the 50% recall rate, combining the search orderings gave a precision of 57% at the Ecdysozoa node and a precision of 50% at the Animal node. Among the top 25 genes returned by the MSGR, the Animal node had the highest recall rate (9/12), which was higher than that of the single organism results, including human (11/49), worm (3/9), and fly (3/10) (see Table 2).

To obtain evidence that the search engine identified genes belonging to a coherent functional group, we inspected the top 25 genes returned by the MSGR (see Table 3). Several genes had functions related to cell-adhesion processes. Among the 16 non-query genes identified were both the *α *and *β *subunits of the non-erythrocytic spectrin, which interacts with cell-adhesion molecules to form cell-attachment sites [26]; crystallin alpha-2, which is involved in structural integrity of eye cells and has been implicated in the structural integrity of neurons [27] and muscle cells [28]; and adiponectin, whose protein sequence contains a conserved collagen-like domain and is highly expressed in muscle cells [29]. Six of the remaining 12 genes also had cytoskeletal roles (*P *< 1E-11), including limatin, syntaxin, filamin, contactin, integrin, and procollagen-lysine 2-oxoglutarate 5-dioxygenase 3.

Six of the 25 genes had no known direct involvement in cell adhesion. These genes were ataxin 2, high density lipoprotein binding protein (vigilin), AMPK catalytic subunit family member 5 (ARK5), aminoadipate-semialdehyde synthase, ATPase H+ transporting lysosomal accessory protein 1, and calumenin. These findings provide additional evidence to link two of these genes to cytoskeletal roles: the ataxin 2 gene associates with T- and L-plastins, which belong to a highly conserved actin binding family [30], and calumenin is a Ca^2+^-binding protein of unknown function that is upregulated during bone healing [31]. These genes may be good candidates for future studies to further characterize their relationship to cell adhesion (for a list of the 500 top-scoring genes see Additional file 9, Supplemental Table 4).

To test the hypothesis that the genes returned by the MSGR participate in a core cell-adhesion pathway, we searched for potential *cis*-regulatory control sequences in the hit list returned by the MSGR (see Methods). The genes returned by the MSGR for the *Collagens *pathway shared binding sites for transcription factors that regulate members of the collagen family. The upstream sequences of these genes were significantly enriched for motifs corresponding to binding sites recognized by the early growth response family of transcription factors *EGR1*, *EGR2*, and *EGR3*. *EGR1 *is a structural gene regulator that increases transcription levels of the *α*1 and *α*2 chains of type I collagen and the a1 chain of type II collagen [32]. We found upstream sequences for 23 of the top 25 scoring genes in the hit list. Our transcription factor analysis identified 14 putative EGR-family transcription factor binding sites (either *EGR1 *or *EGR*) in 10 of these 23 genes. Nine genes had hits to the more general *EGR *binding site. Based on random simulation (see Methods), we found this to be significantly more than the 3.6 genes expected by chance (*P *< 0.001, see Additional file 10, Supplemental Table 5). When the mean score across all genome-wide upstream sequences was used as the cutoff, six additional genes were found to have hits (see Additional file 12, Supplemental Table 7). Thus, 16 of the 23 genes may be regulated by EGR-family transcription factors.

One gene from the original query set, COL5A1, was excluded by the MSGR algorithm. This gene did not have a hit to either the EGR or EGR1 binding site. This finding is consistent with the MSGR's prediction that COL5A1 is under different regulatory control than the other collagen-related query genes.

To determine if the identified binding motifs localize to areas of high sequence conservation, we used the UCSC Genome Browser's *PhastCons *track [33] to estimate the probability of conservation for each nucleotide in the human genome based on an alignment of 17 vertebrate animals. Ten of the 12 regions representing the predicted binding sites had high levels of sequence conservation (see Additional file 13, Supplementary Table 8). The conservation of these sites supports the hypothesis that the high-scoring genes returned by the MSGR belong to a module under common regulatory control by a known cell-adhesion transcription factor.

### Systematic evaluation of the MSGR

**Figure 4:**
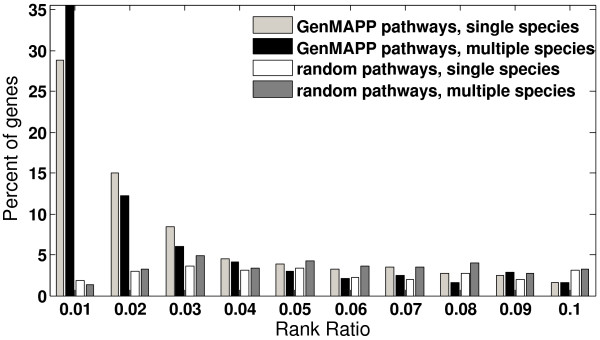
**The MSGR ranks pathway genes higher than genes from random pathways**. Each bin counts the percent of genes belonging to a pathway that sorted to a particular position in the search ordering. A rank-ratio of 0.10 is equivalent to the 90^th ^percentile; a rank-ratio of 0.01 is equivalent to 99^th ^percentile. Bars correspond to GenMAPP pathways run through single-species PMT nodes (dark gray) or multiple-species ancestral PMT nodes (black) and to random pathways run through single-species nodes (white) or multiple-species ancestral nodes (light gray).

Next, we tested the MSGR on a large collection of pathways from the GenMAPP database [12]. To measure its specificity, we compared the search engine's performance on GenMAPP to randomly constructed pathways that matched the sizes of the GenMAPP pathways. Genes unrelated to a pathway should be assigned rank-ratios that are uniformly distributed between 0 and 1. We cross-validated both the GenMAPP pathways and the random pathways and plotted the rank-ratios. The MSGR ranked genes from known pathways closer to the top of its hit lists than genes from random pathways (see Figure 4). The rank-ratios from the GenMAPP pathways were shifted significantly to the left (toward smaller rank-ratios) while the random pathways were distributed more uniformly. For example, 15 times more query genes from GenMAPP pathways were in the top 1%, with rank-ratio less than 0.01, than from random pathways.

**Figure 5:**
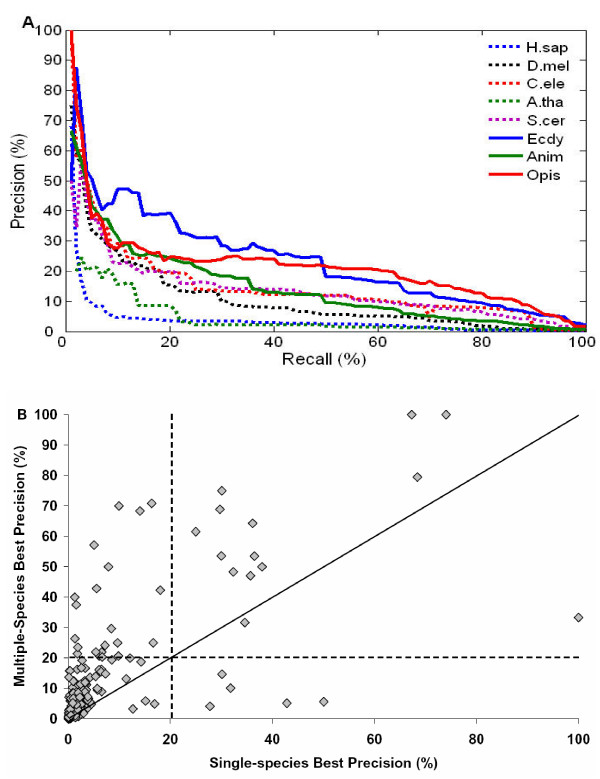
**Accuracy of single- versus multiple-organism searches**. **A. **Precision, averaged across all GenMAPP pathways, is plotted against different levels of recall. The results obtained for individual PMT nodes are shown as separate lines with single-organism results shown as dashed lines and multiple-organism node results shown as solid lines. Average precisions for *A. thaliana*, *H. pylori*, Eukaryote, and Cellular nodes are not shown because each had fewer than 10 pathways with the minimum number of query genes. **B. **The highest precision level at 50% recall rate for all single-organism nodes versus multiple-organism nodes. Each dot corresponds to the precision obtained by the search-engine for a single pathway. Solid line indicates *y = x*, the line of equal performance; multiple-organism searches resulted in higher precision for pathways above the line and a lower precision for pathways below the line. Dashed lines indicate a high precision rate of 20%.

To systematically compare the precision achieved in multiple- versus single-species searches, we evaluated the MSGR's performance on every pathway that had at least five query genes available for searches in single- and a multiple-species. For each pathway, the average precision across the GenMAPP pathways at the 50% recall rate at each of the 11 nodes in the PMT was plotted (see Figure 5A and Methods). At the 50% recall rate, the Opisthokont node achieved the highest precision (31%), followed by the Ecdysozoa node (22%), and finally the Yeast node (18%). The combination of a set of organisms yielded a higher average precision than any single organism over the majority of recall levels tested. For example, the precision-recall curve for the Ecdysozoa node was always above the curves for the Fly and Worm nodes.

We then asked whether the precision levels of the multiple-species search orderings were significantly higher than those of the single-species searches when all levels of recall were considered. The area under the precision recall curve was computed for each pathway, and the means and standard deviations of these areas for each search node across the pathways was determined. The precision of search orderings of Ecdysozoa and Opisthokont nodes was statistically higher than the precision of the corresponding single-species search nodes (see Additional file 2, Supplemental Figure 1). In other cases, the precision of the multiple-species search was statistically comparable to that of the single-species searches (e.g., Worm versus Animal search orderings). In such cases, not enough statistical power is available to conclude that the multiple-species search is more precise. Using a larger collection of pathways might provide a clearer separation in these cases. When the best precision achieved by any of the multiple-species nodes was compared to the best of the single organism nodes for each pathway, the majority of pathways had higher precision for a multiple-species node. (see Figure 5B; most points lie above the line of equal performance).

The higher performance of the multiple-species searches was robust to several factors. To determine if the particular datasets used in the search significantly affected the *Collagens *search result, we removed 5 – 90% of random microarray hybridizations for each organism. The *Collagens *search orderings were highly reproducible. For example, even when 90% of the data was withheld, the search engine found 20 – 60% of the original top 50 hits (see Additional file 3, Supplemental Figure 2). To determine if the higher precision of the multiple-species searches than the single-species searches simply reflected the use of conserved query genes, we repeated the cross-validation analysis for the Human node using only query genes that had predicted orthologs in both worms and flies. The precision obtained by the Human node using only conserved query genes was comparable to that obtained using the full set of query genes, suggesting that the improvement observed in the MSGR search is not simply due to restricting query genes to those genes that are highly conserved (see Additional file 4, Supplemental Figure 3).

Finally, to determine if the search results were sensitive to errors in the orthology prediction, we randomly shuffled a fraction of the orthology mapping, reran the MSGR on the 39 GenMAPP pathways, and recorded the average precision for every level of average recall at the Ecdysozoa node. While the accuracy of the search does rely on a good orthology prediction, the search orderings had precision levels comparable to those of single-species searches, even when as many as 25% of the orthology assignments were shuffled. Shuffling more than 25% of the orthology assignments led to decreased performance when searches were combined across species (see Additional file 5, Supplemental Figure 4).

### Global prediction of new pathway members

We used the MSGR to identify pathways that were significantly coregulated in datasets from two or more organisms, as these are the most promising for identifying new candidates. In addition to the GenMAPP pathways, we ran the MSGR on pathways from Gene Ontology [13], BioCarta [34], and KEGG [14]. Altogether, we collected a total of 553 pathways for our search (see Additional file 6, Supplemental Table 1).

**Figure 6:**
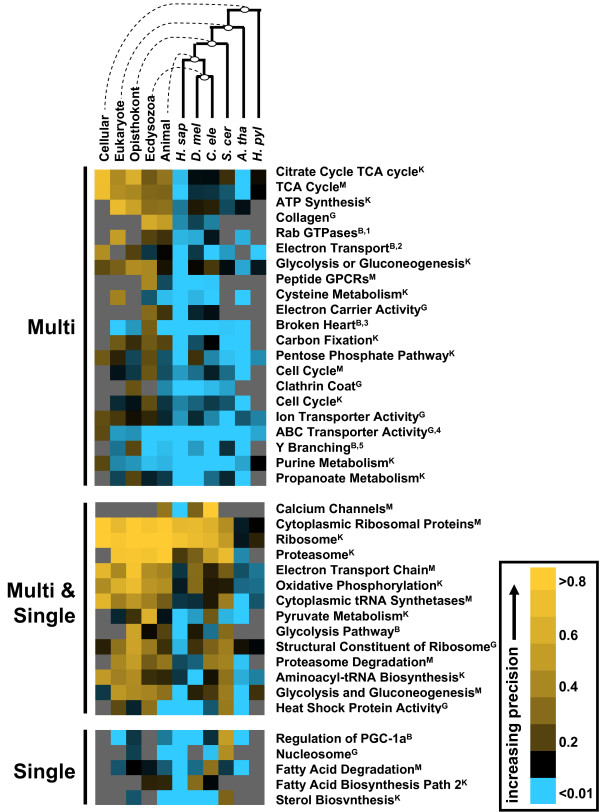
**Pathways with precise search results**. The precision obtained at every PMT search node is shown for pathways that achieved a precision of at least 20% in one or more nodes of the PMT at the 50% recall rate. Yellow corresponds to high precisions near 100%, and blue to low precisions near 0%. The first 21 rows (Multi) correspond to pathways for which a precision of at least 20% was achieved only by a multiple-organism node. The next 14 rows (Multi & Single) correspond to pathways for which both multiple- and single-organism nodes achieved a precision of at least 20%; the last five rows (Single) correspond to pathways for which only a single-organism node achieved a precision of at least 20%. Superscript letters indicate the database from which pathways were collected: K, KEGG [14]; G, GO [13]; M, GenMAPP [12]; B, BioCarta [34]. Superscript numbers indicate pathway names that were abbreviated: 1, *Rab GTPases Mark Targets in the Endocytotic Machinery*; 2, *Electron Transport Reaction in Mitochondria*; 3, *NFAT and Hypertrophy of the Heart Transcription in the Broken Heart*; 4, *ATP-Binding Cassette ABC Transporter Activity*; 5, *Y Branching of Actin Filaments*.

In general, the searches combining multiple organisms had higher precision levels for more pathways than the searches conducted on single organisms. The result of cross-validation identified 40 pathways with high precision (at least 20% at the 50% recall rate) in at least one organism (see Figure 6, and Additional file 14, Supplemental Table 9 for the full set of predictions). Over half (21) of these pathways achieved high precision when searches from multiple species were combined even though no single species achieved high precision; 14 had high precision in both multiple- and single-species (see Figure 6). Of the 19 pathways with high precision in one organism, 74% (14) also had high precision in multiple organisms. Thus, the expression data appear to have an appreciable degree of complementary information useful for cross-species inferences about gene function.

Pathways with high precision at multiple nodes in the PMT correspond to highly conserved core processes. As expected, several pathways related to the ribosomal subunits and ribosome assembly ranked at the top of the list, as did pathways related to protein degradation, energy generation (e.g., ATP synthesis, TCA cycle, and glycolysis), and stress response. On the other hand, only five pathways achieved higher precision with results from a single organism than with results from multiple species. These may represent cases where experiments relevant to the pathway were collected only on a single organism or where pathways diverged in a lineage-specific fashion so that different sets of genes are coregulated with one another across the organisms.

## Discussion

Combining search orderings from multiple organisms into a single search ordering using the MSGR algorithm improved our ability to predict gene function for several pathways. The MSGR ranked more query genes to the top of the search orderings compared to the search orderings returned by the GeneRecommender on the same GenMAPP pathway. Overall, the precision levels were statistically higher for multiple-species searches than for single-species searches. In most cases where a search in an organism yielded a highly precise result for a pathway, the performance could be improved by combining results from at least one other organism, The results were relatively insensitive to the particular experiments included in the gene expression database and to small levels of error in the orthology predictions.

The MSGR predicted genes for multifunctional pathways with high precision. For example, combining search orderings across the multicellular animals significantly improved the predictive power for finding genes involved in the *Collagens *pathway. The genes returned in the *Collagens *search may represent a conserved cell-adhesion module. Several genes returned by the search engine were not known to function with genes in the query and yet shared *cis*-regulatory sequences with high-scoring query genes. The gene that encodes calumenin was a high-scoring hit for the *Collagens *query and contains a putative binding site for the collagen-inducing factor encoded by the *EGR1 *gene. These results suggest specific follow-up experiments to test calumenin's association with the collagens. For example, one could conduct a chromatin-immunoprecipitation assay (ChIP-chip) in which *EGR1 *binding affinity to the upstream region of calumenin is measured. Other high-scoring genes returned by the MSGR could also be similarly tested using a DNA microarray in conjunction with the chromatin-immunoprecipitation assay.

The higher performance of the combined searches than of single-species searches may reflect the MSGR's ability to focus on gene activity that is functionally relevant. Coregulation of a pair of genes conserved across large evolutionary distances implies that the coregulation confers a selective advantage. The meta-search strategy implemented by the MSGR may be making use of the presence of biologically relevant couplings between such genes.

While the MSGR has shown initial promise as a predictive tool, we anticipate future improvements that address several of the MSGR's current limitations. The MSGR has a limited view of gene function because it uses gene expression data exclusively. Researchers can now access other kinds of genome-scale datasets such as protein – protein interaction maps [35] and genome-wide RNAi screens [36]. However, since the MSGR uses only the ranks from different searches, the results of a search engine for a new data type can be incorporated into the MSGR. We plan to investigate which combinations of data sources improve the performance for specific pathways.

The MSGR is also limited because it attempts to use the entire input pathway without modification. To circumvent this limitation, an algorithm that iteratively adds to and subtracts from the current query set could be implemented. High-scoring genes from one round could be included as query genes in the next round, in an approach analogous to that used by the Signature Algorithm [6]. A given query could also be split into functionally distinct subgroups of genes in a pathway. Genes in each subpathway may be more tightly coregulated with each other than with other genes in the pathway. In such cases, the query set could be partitioned by running cross-validation to score each held-out query gene. High-scoring query genes could then be retained as the new set of query genes. Repeating this procedure on the remaining unselected queries might identify tightly coupled subqueries for which separate recommendations could be provided.

Finally, the MSGR uses an orthology prediction based on reciprocal BLAST comparisons of peptide sequences to link gene expression data across species. The MSGR takes a conservative approach by using only BTPs where no conflicts are present. Because some protein families are large, this approach cannot resolve the orthology assignments for many human genes, significantly reducing the number of genes that can be used as queries. Adding organisms that are more closely related to human, such as mouse, will help to increase this number for human pathway searches. A future direction is to test alternative orthology prediction sets with the MSGR such as INPARANOID [37] and OrthoMCL [38].

## Conclusion

The molecular biology community continues to amass data about complex genetic pathways by using high-throughput technologies. It is critical to the progress of both science and medicine that, in addition to the organization and dissemination of this information, methods for searching the data be developed to further improve our understanding of genetic pathways. Our approach aims to help biologists broaden their understanding of molecular mechanisms by enabling them to quickly scan for new genes that are coregulated with a query list of genes. The MSGR search engine should allow functional genomics data to be tapped for investigations of a broad range of genetic mechanisms. A user interface and the source code for the MSGR are available from our website [39].

## Materials and methods

Given a set of genes that are functionally related, called the *query*, the MSGR scans gene expression data across multiple organisms to identify genes of related function. The MSGR combines search orderings from searches in multiple organisms into a single search ordering. The MSGR merges the search orderings using a probabilistic scoring method based on order statistics and outputs a ranked list of genes sorted by their scores. Genes that rank toward the top of independent GeneRecommender searches in different organisms receive smaller *P *values (more significant scores) than genes with lower ranks across the organisms.

### Gene expression datasets

For this study, we incorporated data from six organisms: *Homo sapiens*, *Drosophila melanogaster*, *Caenorhabditis elegans*, *Saccharomyces cerevisiae*, *Arabidopsis thaliana*, and *Helicobacter pylori *(henceforth be referred to as human, fly, worm, yeast, plant, and bacterium, respectively). Gene expression data from Gene Expression Omnibus was collected on July 17, 2004, and were combined with the data from our previous study [40]. The combined data reflect 5692 microarray hybridization measurements (referred to as experiments) in humans, 334 in flies, 1209 in worms, 753 in yeast, 649 in plants, and 293 in bacteria. The number of genes probed was 22,080 in humans, 13,403 in flies, 21,925 in worms, 6330 in yeast, 9169 in plants, and 1590 in bacteria.

All single- and dual-channel hybridization results were compiled into a single compendium for each organism. A log-ratio for the single channel results was calculated before merging with the dual-channel results. We first collected all of the single channel data and computed a virtual reference for every gene as the median expression value of the gene across all of the collected single channel arrays. We then divided each absolute single channel value by the gene's virtual reference and took the logarithm base-two. The dataset for each organism is available from our website [39].

### Gene pathway datasets

We collected a total of 553 human genetic pathways from several pathway databases on August 16, 2004. These included 106 human pathways from KEGG [14], 51 from the GenMAPP collection [12], 257 from BioCarta [34], and 139 from Gene Ontology [13] (GO). To define a nonredundant set of pathways from the GO database, we restricted the list of the total 5399 categories to those that occur at level three in the GO hierarchy and that contained between five and 300 genes (see Supplemental Table 1 for a complete list of all of the pathways used in this study). Some of the pathways are very similar. For example, three categories represented the cytosolic ribosome: the *Ribosome *KEGG category, the *Cytoplasmic Ribosomal Proteins *GenMAPP category, and the *cytosolic ribosome (sensu Eukarya) *GO category. We kept this redundancy in our pathway lists because the lists in the different databases often differed, even for well-documented pathways. For example, the proteasome category in the KEGG database *Proteasome *had 30 genes, while the GO category *proteasome complex (sensu Eukarya) *had 35 genes. Because such differences could be important for predicting gene function, we chose to be inclusive and investigate all pathways. Each gene in a pathway was associated with data in our expression database by using each gene's corresponding EntrezGene identifier [41]. A summary of the number of genes and the number of pathways is listed in Table 4.

### Search procedure

The MSGR takes as input a set of genes, the *query*, and calls the GeneRecommender [7] to find genes that are highly coregulated with the query genes. The expression data for species *s *is normalized so that each gene's expression levels are ranked and uniformly arranged on the interval (-1,+1). The GeneRecommender uses the normalized matrix of expression levels to identify informative experiments and then rank genes according to their correlation with the query in those experiments.

### Identifying informative experiments

A set of query genes may be coactivated in only a subset of the experiments in a database. We expect these experiments to be informative in the sense that they are likely to be the most useful experiments for identifying additional genes with shared function. To identify informative experiments, the GeneRecommender scans for microarray experiments in which the given query genes are either coordinately up- or down-regulated. It scores the *e*^th ^experiment in species *s *using the following score:

E(e,s)=kesμQesσQes2+13ms2,
 MathType@MTEF@5@5@+=feaafiart1ev1aaatCvAUfKttLearuWrP9MDH5MBPbIqV92AaeXatLxBI9gBaebbnrfifHhDYfgasaacH8akY=wiFfYdH8Gipec8Eeeu0xXdbba9frFj0=OqFfea0dXdd9vqai=hGuQ8kuc9pgc9s8qqaq=dirpe0xb9q8qiLsFr0=vr0=vr0dc8meaabaqaciaacaGaaeqabaqabeGadaaakeaacqWGfbqrcqGGOaakcqWGLbqzcqGGSaalcqWGZbWCcqGGPaqkcqGH9aqpdaGcaaqaaiabdUgaRnaaBaaaleaacqWGLbqzcqWGZbWCaeqaaaqabaGcdaWcaaqaaGGaciab=X7aTnaaBaaaleaacqWGrbqucqWGLbqzcqWGZbWCaeqaaaGcbaWaaOaaaeaacqWFdpWCdaqhaaWcbaGaemyuaeLaemyzauMaem4CamhabaGaeGOmaidaaOGaey4kaSYaaSaaaeaacqaIXaqmaeaacqaIZaWmcqWGTbqBdaqhaaWcbaGaem4CamhabaGaeGOmaidaaaaaaeqaaaaakiabcYcaSaaa@4D05@

where *k*_*es *_is the number of query genes with valid data in experiment *e*, *μ*_*Qes *_is the mean, *σ*_*Qes *_is the standard error of the normalized expression levels of the query genes, and *m*_*s *_is the number of total experiments in species *s*. Extreme values of *E(e, s) *indicate that an experiment may help predict new genes because several of the query genes obtain either highly positive or highly negative expression levels relative to the rest of the experiments in the database. The GeneRecommender selects a set of experiments, *I*, with absolute scores exceeding an optimized cutoff, *c*. To optimize the cutoff, the GeneRecommender tries several different values and chooses the threshold that gives the highest precision at the 50% recall rate.

### Ranking genes by relevance

The GeneRecommender scores every gene *g *based on the extent to which its expression profile correlates with the query over the informative experiments identified in the previous step. The GeneRecommender's score for gene *g *is:

G(g,s)=∑e∈IμQesXges13∑e∈IμQes2,
 MathType@MTEF@5@5@+=feaafiart1ev1aaatCvAUfKttLearuWrP9MDH5MBPbIqV92AaeXatLxBI9gBaebbnrfifHhDYfgasaacH8akY=wiFfYdH8Gipec8Eeeu0xXdbba9frFj0=OqFfea0dXdd9vqai=hGuQ8kuc9pgc9s8qqaq=dirpe0xb9q8qiLsFr0=vr0=vr0dc8meaabaqaciaacaGaaeqabaqabeGadaaakeaacqWGhbWrcqGGOaakcqWGNbWzcqGGSaalcqWGZbWCcqGGPaqkcqGH9aqpdaWcaaqaamaaqafabaacciGae8hVd02aaSbaaSqaaiabdgfarjabdwgaLjabdohaZbqabaGccqWGybawdaWgaaWcbaGaem4zaCMaemyzauMaem4CamhabeaaaeaacqWGLbqzcqGHiiIZcqWGjbqsaeqaniabggHiLdaakeaadaGcaaqaamaalaaabaGaeGymaedabaGaeG4mamdaamaaqafabaGae8hVd02aa0baaSqaaiabdgfarjabdwgaLjabdohaZbqaaiabikdaYaaaaeaacqWGLbqzcqGHiiIZcqWGjbqsaeqaniabggHiLdaaleqaaaaakiabcYcaSaaa@5563@

where *X*_*ges *_is the normalized expression level of *g *in the *e*^th ^experiment of species *s*. *G(g, s) *is a dot-product between gene *g*'s expression profile and the centroid of the query genes scaled by an estimate of the standard deviation of *g*'s expression levels. The MSGR sorts all of the genes in decreasing order by *G(g, s) *and assigns a rank, *v*_*gs*_, corresponding to *g*'s rank in the search ordering of species *s*. To compute a combined score for the gene, the MSGR uses the ranks obtained for a gene and its orthologs across the six species, *(v*_*g1*_, *v*_*g2*_, ..., *v*_*g6*_*)*.

### Predicting orthologous genes

The query set may contain genes from any one of the six organisms, which is referred to as the *source *organism. To scan gene expression datasets from additional organisms other than the source organism, the MSGR translates the query genes from the source organism to a *target *organism by using a sequence-similarity look-up table.

The look-up table containing putative orthologous relations is constructed by finding all best protein matches (BPMs) for every gene in each of the six organisms. A BPM for each gene from a source organism is found by using the BLASTP algorithm [42] to compare the gene product's amino acid sequence to that of each gene product in a target organism. A BPM is an ordered pair *(g, h) *recording which gene *h *in the target organism has the best matching peptide sequence to gene *g *from the source organism. Gene *h *is found by identifying the gene with the minimum E-value among all of the genes in the target organism's protein sequence database. Matches with E-values greater than 1E-5 are excluded from consideration.

In many cases, multiple genes from the source organism match the same gene in the target organism. These situations may be the product of gene duplication events, giving rise to one gene that retained the ancestral function and another gene that diverged in function. To avoid matching paralogous genes from a source organism, we include only the highest-scoring gene from a source organism. Define the best target protein (BTP) for the source query gene *g *as the gene *h *in the target organism where *(g, h) *either is the only BPM for gene *h *or has the highest BLASTP score of all BPMs in the source organism that include gene *h*. BTPs for all genes in the query are identified and used as the query for the target organism. Genes that lack a BTP in a species are excluded from the search ordering in that species. The table of all orthologous assignments can be found on our website (see Additional file 15 Supplemental Table 10). Note that the BTP defined here is not strictly reciprocal since gene *g *may not be the BTP of gene *h* .

### Combining search orderings across organisms

Given a query, the MSGR scores each gene in the genome based on how similar its expression levels are to the expression levels of the query genes. The MSGR is run on the six organisms individually, yielding independent search orderings for each organism having at least the minimum number of genes orthologous to those genes in the query (five by default). To construct a query set in fly from a query set in human, orthologous protein sequences in fly are identified by looking for fly proteins associated with the given human proteins in the BTP table that translates human proteins to fly proteins. The GeneRecommender is used to rank all of the genes in a single organism's genome in decreasing order of expression similarity to the query set. The expression similarity is computed over a set of informative conditions identified by the GeneRecommender algorithm.

As illustrated in Figure 3, there are five branches in the phylogenetic merge tree (PMT), corresponding to the five common ancestors shared by the six organisms: the cellular ancestor common to all of the organisms, the eukaryotic ancestor common to all of the organisms except the bacterium *H. pylori*, the opisthokont ancestor common to the non-plantlike eukaryotes, the animal ancestor of the multicellular eukaryotes, and the ecdysozoan ancestor of the molting organisms from which flies and worms descended. Nodes in the PMT are referred to using labels containing an initial uppercase letter: Human, Fly, Worm, Yeast, Plant, Bacterium, Cellular, Eukaryote, Opisthokont, Animal, and Ecdysozoa. For any given query, 11 different search orderings are returned corresponding to the six searches run on the individual organisms plus the five combined search orderings at the ancestral nodes of the tree.

The MSGR combines the organism-specific search orderings to produce a new search ordering for each ancestral node in the PMT. Fly and Worm orderings are merged into a new search ordering at the Ecdysozoa node. Human, Worm, and Fly orderings are merged into a single ordering at the Animal node. The search orderings of all six organisms are combined to produce a new search ordering at the Cellular node. A gene is included in the search ordering of an ancestral node if a BTP exists in the search ordering of at least one organism in both the left and right sub-trees beneath the node. For example, a gene will be used at the Cellular node if its orthologs appear in search orderings in either Human, Fly, Worm, Yeast, or Plant nodes and a search ordering in the Bacterium node.

The result of an MSGR search at a particular node *t *in the PMT associates a set of ranks to a particular candidate gene. Recall that *v*_*gs *_is the rank of candidate gene *g *in the search ordering of species *s*. Each rank is converted to a rank-ratio, *r*_*gs *_= *(v*_*gs *_- *1)/(N*_*s *_- *1)*, where *N*_*s *_is the maximum rank possible for species *s*. The rank-ratios range between 0, indicating *g *is coregulated with the query, and 1, indicating *g *is not coregulated with the query. The rank-ratios for *g *at node *t *are recorded in increasing order in the list denoted *R*_*gt*_. For example, at the Animal node, *g*'s ranks might be *R*_*gAnimal *_= *(r*_*gHuman*_,*r*_*gFly*_,*r*_*gWorm*_*) *if *r*_*gHuman *_≤ *r*_*gFly *_≤ *r*_*gWorm *_and *g *had orthologs with expression data in these three organisms.

The rank-ratios assigned to *g *at node *t *are scored to reflect their degree of non-randomness. The *P *value for the probability of observing a set of *n *rank-ratios or smaller by chance is computed. Small *P *values correspond to candidates that are more likely to be related to the query pathway. For a gene that is unrelated to the original query set, its rank-ratios are expected to be uniformly and independently distributed between 0 and 1. Thus, the *P *value can be computed from the joint cumulative distribution of a set of uniformly distributed order statistics. The following recursive formula gives this quantity:

P(Rgt)=∑j=1|Rgt|(Rgt[j]−Rgt[j−1])P(Rgt[−j]),
 MathType@MTEF@5@5@+=feaafiart1ev1aaatCvAUfKttLearuWrP9MDH5MBPbIqV92AaeXatLxBI9gBaebbnrfifHhDYfgasaacH8akY=wiFfYdH8Gipec8Eeeu0xXdbba9frFj0=OqFfea0dXdd9vqai=hGuQ8kuc9pgc9s8qqaq=dirpe0xb9q8qiLsFr0=vr0=vr0dc8meaabaqaciaacaGaaeqabaqabeGadaaakeaacqWGqbaucqGGOaakcqWGsbGudaWgaaWcbaGaem4zaCMaemiDaqhabeaakiabcMcaPiabg2da9maaqahabaGaeiikaGIaemOuai1aaSbaaSqaaiabdEgaNjabdsha0bqabaGccqGGBbWwcqWGQbGAcqGGDbqxcqGHsislcqWGsbGudaWgaaWcbaGaem4zaCMaemiDaqhabeaakiabcUfaBjabdQgaQjabgkHiTiabigdaXiabc2faDjabcMcaPiabdcfaqjabcIcaOiabdkfasnaaBaaaleaacqWGNbWzcqWG0baDaeqaaOGaei4waSLaeyOeI0IaemOAaOMaeiyxa0LaeiykaKcaleaacqWGQbGAcqGH9aqpcqaIXaqmaeaacqGG8baFcqWGsbGudaWgaaadbaGaem4zaCMaemiDaqhabeaaliabcYha8bqdcqGHris5aOGaeiilaWcaaa@62C0@

where *R*_*gt *_is the set of ordered rank-ratios, *P(R) *is the probability of getting rank-ratios equal to or smaller than the ordered rank-ratios in *R*, *R[j] *is the j^th ^largest rank-ratio in *R*, and *R [-j] *is the set of rank-ratios remaining after removing the *j*^*th *^largest rank-ratio (a proof demonstrating the correctness of the formula is included in the Appendix). This is an approximation because the rank-ratios are discrete rather than continuous. However, because the expression datasets contain many genes, the continuous approximation to the discrete distribution is expected to be sufficiently accurate.

### Measuring the precision and recall of a search

The accuracy of a search is assessed with a cross-validation test in which a measure of precision and recall is computed based on the MSGR's ability to rank withheld members of the pathway toward the top of the returned search ordering. Given a query *Q *of *n *genes, we randomly partition the set of genes into five folds. Each fold *f *holds out a set of genes *H*_*f *_from the query and reconstructs a new query set, *Q*_*f*_, which has ⌊4*n/5*⌋ genes, *Q*_*f *_= *Q *- *H*_*f*_. The rank percentiles for each left-out gene in *H*_*f *_are collected from all of the folds to obtain ranks for all of the original query genes.

The number of pathway genes *a *and nonpathway genes *b *that receive a rank of *c *or better when withheld in a particular cross-validation fold are counted. By varying the cutoff *c*, we obtain different values for *a *and *b*. The precision is then computed as *a/(a+b) *which reflects the degree to which the query genes rank toward the top of the list in a particular cross-validation fold. The recall is computed as *a/k*, where *k *is the total number of pathway genes that were scored by the MSGR. The average of the precision and recall is taken across the cross-validation folds to obtain an average precision and an average recall for a single pathway. Note that the value of *k *changes depending on which PMT node is being searched. For example, some genes may not have a BTP or may not have any data collected in any one of the organisms beneath a particular node in the PMT. A range of average precisions as a function of the average recall is obtained by sweeping through different values of *c*.

### Identifying overrepresented transcription factor binding sites in a hit list

Putative binding sites that are significantly overrepresented in the upstream regions of the genes returned in a hit list returned by the MSGR are identified by scanning matrices representing 341 human binding site models taken from release 7.2 of the TRANSFAC database [43]. Upstream 1000 base-pair regions of 14,368 genes were downloaded from the UCSC Genome Browser [44] for the human genome (May 2004 build) [45].

A binding site score between a gene *g *and matrix *m*, *B*_*gm*_, is computed with *tffind *[46], a program that assigns a score, ranging from 0 (very different from *m*) to 1 (perfect match to *m*), for each position in *g*'s upstream region. *B*_*gm *_is defined as the highest position-specific score. Because different transcription factors may have different binding specificities, it is unknown what cutoff for *B*_*gm *_to use, above which *m *is considered a match to gene *g*. We therefore try a series of cutoffs to identify which cutoff gives the optimal separation between the genes in the hit list and all of the genes in the genome. For each *m*, the cutoff is varied between *α*_*m *_and 1, where *α*_*m *_is the mean of the best score attained by the matrix across all upstream regions in the genome. For each cutoff, the percentage of background genes that have a best score at least as high as the cutoff is recorded. This value is then used to estimate the *P *value for the match between *m *and the hit list using the hypergeometric distribution. The cutoff that achieves the best *P *value is chosen as *c*_*m *_and used to determine which genes in the hit list have a hit to the matrix. All matrices with a *P *value of 0.01 or lower and with at least 25% coverage of the genes in the hit list are reported. We also report an *enrichment score*, defined as the ratio *h/e*, where *h *is the percent of genes in the hit list that have a match to *m *and *e *is the percent of genes in the background distribution that have a hit to *m*.

The significance of the number of genes in a hit list with matches to binding site matrix *m *is estimated by using a simulation to construct a null distribution for *m*. *k *genes are drawn randomly from the genome, where *k *is the same size as the number of genes in the hit list. Each gene's upstream region is scored with matrix *m *by counting the number of randomly drawn genes that achieve scores equal to or greater than *c*_*m*_. A distribution is constructed from 1000 random simulations. To obtain the corresponding *P *value for that matrix, the number of matches to matrix *m *in the hit list is compared to the mean and standard deviation of this distribution.

### Determining the conservation of binding site predictions

The degree of conservation of a predicted binding site was determined with the *PhastOdds *program [33] and the conservation track of the UCSC Genome Browser [33] (May 2004 build) [33]. This build contains multiple alignments of 17 vertebrates, including mammals, amphibians, birds, and fish. A log-odds conservation score for each binding site prediction was determined with *PhastOdds*. The *PhastOdds *score is a ratio of the likelihood of two phylogenetic models: a positive model to detect conserved sequences and a background model representing nonconserved sequences. Regions with scores greater than 0 are considered conserved because the observed conservation patterns fit the conserved model better than the nonconserved model. The nonconserved model was estimated from fourfold degenerate sites in coding regions using the *PhyloFit *[33] program. The positive model was derived from the nonconserved model except that the model's substitution rate parameter was estimated using *PhastCons *[33] run on multiple alignments of the 17 vertebrates produced by the ENCODE Consortium [47]. Before running the conservation analysis, overlapping predictions are merged to avoid over-estimating the number of conserved binding sites.

## Authors' contributions

CC implemented the algorithm and performed the computational tests. MW performed the transcription factor binding analysis and helped to draft the manuscript. CP developed and wrote the mathematical proofs. AZ participated in the analysis of the search results. JS conceived of the study, and participated in its design and coordination and drafted the manuscript. All authors read and approved of the final manuscript.

## Supplementary Material

Additional file 1 Appendix. Discussion of related work and proof of correctness of order statistics implementation. Click here for file

Additional file 7 Table S2. The query list of genes for the *Calcium Channels *and *Collagens *GenMAPP pathways. Click here for file

Additional file 10 Table S5. Summary of significant *cis*-regulatory motifs identified in the *Collagens *and *Calcium Channels *search results. Click here for file

Additional file 11 Table S6. Sequences of potential *cis*-regulatory binding sites of transcription factors identified in the *Calcium Channels *search results. Click here for file

Additional file 12 Table S7. Sequences of potential *cis*-regulatory binding sites identified in the *Collagens *search results. Click here for file

Additional file 13 Table S8. Conservation of predicted binding sites in the *Collagens *search result. Click here for file

Additional file 2 Figure S1. Precision-recall results of the MSGR run on the GenMAPP test pathways. Plotted are the same results as shown in Figure 5A of the text, but the precision estimates have here been augmented to include standard error bars to show the variability of the MSGR's performance across the pathways. Click here for file

Additional file 3 Figure S2. Robustness of the Collagens search result to various fractions of held-out data. Click here for file

Additional file 4 Figure S3. Performance of single-species search using query sets restricted to conserved genes. Click here for file

Additional file 5 Figure S4. Performance as a function of orthology prediction error. Click here for file

Additional file 6 Table S1. The pathways tested in the study. Click here for file

Additional file 14 Table S9. Pathways for which accurate predictions with the MSGR could be made. Click here for file

Additional file 8 Table S3. The top 500 genes returned by the MSGR search at the Animal node for the *Calcium Channels *GenMAPP pathway. Click here for file

Additional file 9 Table S4. The top 500 genes returned by the MSGR search at the Animal node for the *Collagen *GenMAPP pathway. Click here for file

Additional file 15 Table S10. List of putative orthologs used in the study. Click here for file

## Figures and Tables

**Table 1:** Top 25 genes returned by the Animal PMT node using the GenMAPP *Calcium Channels *pathway as a query. The horizontal line indicates the 50% recall point, above which half of the query genes are included.

Gene Product^a^	Symbol	*P*-value^b^	S^c^	N^d^	Z^e^	M^f^	B^g^
*Calcium channel, voltage-dependent, beta 2*	CACNB2	7.6E-10	y^h^	y			y
*Calcium channel, voltage-dependent, L type, alpha 1D*	CACNA1D	3.1E-08	y	y	y	y	y
*Calcium channel, voltage-dependent, P/Q type, alpha 1A*	CACNA1A	1.4E-07	y	y	y	y	
Regulating synaptic membrane exocytosis 2	RIMS2	1.6E-07	y	y			y
MAP-kinase activating death domain	MADD	2.5E-07	y			y	
Contactin 1	CNTN1	6.2E-07	y	y		y	
Glutamate decarboxylase 1	GAD1	7.2E-06	y	y	y	y	
*Ryanodine receptor 2*	RYR2	1.2E-05	y	y	y		

HIVI enhancer binding protein 1	HIVEP1	1.3E-05	y		y	y	
Membrane metallo-endopeptidase	MME	1.8E-05	y	y	y	y	
Stonin 2	STN2	2.0E-05	y	y	y		y
Cholinergic receptor, nicotinic, delta	CHRND	2.0E-05	y	y	y	y	
Transforming growth factor, beta 1	TGFB1	2.3E-05	y		y	y	
Spectrin, beta, non-erythrocytic 1	SPTBN1	2.4E-05	y	y	y		
Spectrin, alpha, non-erythrocytic 1	SPTAN1	3.5E-05	y	y		y	
Soc-2 suppressor of clear (*C. elegans*)	SHOC2	4.0E-05				y	
Protein tyr. Phosph., receptor type, N 2	PTPRN2	4.6E-05	y			y	
Potassium channel, subfamily K, member 3	KCNK3	4.7E-05	y	y	y	y	
Neurotransmitter transporter, noradrenalin, member 2	SLC6A2	4.9E-05	y	y	y	y	
Protein tyrosine phosphatase, non-receptor type 4	PTPN4	6.9E-05	y			y	
Ras association (RalGDS/AF-6) domain family 1	RASSF1	7.6E-05	y		y	y	
Troponin T2, cardiac	TNNT2	7.9E-05		y	y		y
Inositol 1,4,5-trisphosphate 3-kinase B	ITPKB	8.7E-05	y			y	
Ubiquitin specific protease 11	USP11	9.1E-05	y				
Titin	TTN	1.0E-04		y			y

^a^The HUGO name for the gene. Genes in the original query set are italicized.

^b^MSGR combined *P *value. This *P *value was not adjusted to reflect multiple testing; a Bonferroni correction could be applied by multiplying each by 11 to account for searches at each node in the PMT.

^c^Involved in cell – cell or intracellular signaling.

^d^Neuromuscular function.

^e^Predicted binding site for the ZIC family of transcription factors.

^f^Predicted binding site for MAZ or MAZR.

^g^Predicted binding site for the BRAIN2 transcription factor.

^h^A "y" indicates that a gene had a positive hit for a particular field.

**Table 2:** Search result ranks for the *Collagens *pathway returned by the MSGR

**Query Gene**	**Human**	**Fly**	**Worm**	**Animal**
Collagen, type IV, alpha 2	15	98	1	1
Nidogen 1	189	10	146	2
Heparan sulfate proteoglycan 2	5994	52	2	4
Collagen, type IV, alpha 5	1218	1	n/a	9
Secreted protein, acidic, cysteine-rich	20	n/a*	24	6
Laminin, beta 1	1588	34	131	8
Laminin, alpha 2	16	158	1728	7
Collagen, type XV, alpha 1	12	2291	1193	17
Laminin, gamma 1	10883	23	96	12
Lumican	6	1049	n/a	29
Dystonin	274	103	n/a	50
Collagen, type V, alpha 1	87	n/a	5551	263

*n/a corresponds to cases where no best target protein could be identified in a particular organism.

**Table 3:** Top 25 genes returned by the Animal PMT node using the GenMAPP *Collagens *pathway as a query. The horizontal line indicates the 50% recall point, above which half of the query genes are included.

**Gene Product^a^**	Symbol	*P*-value^b^	A^c^	E^d^
*Collagen, type IV, alpha 2*	COL4A2	1.25E-09	y^e^	y
*Nidogen*	NID	1.42E-07	y	
Actin binding LIM protein 1	ABLIM1	1.49E-07		
*Heparan sulfate proteoglycan 2*	HSPG2	5.65E-07	y	y
Spectrin, alpha, non-erythrocytic 1	SPTAN1	7.97E-07		y
*Osteonectin*	SPARC	1.16E-06	y	
*Laminin, alpha 2*	LAMA2	3.61E-06	y	
*Laminin, beta 1*	LAMB1	4.89E-06	y	y

*Collagen, type IV, alpha 5 (Alport Syndrome)*	COL4A5	8.22E-06	y	
Ataxin 2	ATXN2	1.01E-05		y
Adipocyte, C1Q and collagen domain Containing	ACDC	1.44E-05		n/a*
*Laminin, gamma 1 (formerly LAMB2)*	LAMC1	1.77E-05	y	y
Syntaxin binding protein 1	STXBP1	1.95E-05		y
Calumenin	CALU	2.24E-05		y
High density lipoprotein binding protein (vigilin)	HDLBP	2.33E-05		
Crystallin, alpha B	CRYAB	2.41E-05		y
*Collagen, type XV, alpha 1*	COL15A1	2.53E-05	y	
ATPase, H+ transporting, lysosomal accessory protein 1	ATP6AP1	2.57E-05		
Procollagen-lysine, 2-oxoglutarate 5- dioxygenase 3	PLOD3	2.89E-05		
Contactin 1	CNTN1	2.92E-05		
AMP-activated protein kinase family member 5	ARK5	2.93E-05		
Integrin, alpha 8	ITGA8	3.13E-05		n/a
Filamin A, alpha (actin binding protein 280)	FLNA	3.22E-05		y
Aminoadipate-semialdehyde synthase	AASS	3.46E-05		
Spectrin, beta, non-erythrocytic 1	SPTBN1	3.49E-05		

^a^The HUGO name for the gene. Genes in the original query set are italicized.

^b^MSGR combined *P *value. This *P *value was not adjusted to reflect multiple testing; a Bonferroni correction could be applied by multiplying each by 11 to account for searches at each node in the PMT.

^c^Involved in cell – adhesion.

^d^Predicted binding site for the EGR family of transcription factors.

*n/a indicates that an upstream region could not be obtained for the given gene.

^e^A "y" indicates that a gene had a positive hit for a particular field.

**Table 4:** The number of genes and pathways available for use with the MSGR search engine for the six organisms and five ancestral combinations.

	Genes w/Ortho^a^	Genes with Data and Ortho^b^	Genes with Data^c^	Pathways^d^
*H.sap*	17366^e^	17366	22080	539
*D.mel*	5834	5697	13403	492
*C.ele*	5070	4174	21925	490
*S.cer*	2470	2451	6630	428
*A.thal*	4346	1893	9169	405
*H.pyl*	325	324	1590	105
Ecdy	-	-	3024	235
Anim	-	-	6847	406
Opis	-	-	2451	199
Euk	-	-	1893	130
Cell	-	-	324	33

^a^Number of genes with BTPs to a human gene.

^b^Number of genes that have BTPs and have expression data.

^c^Total number of genes with available expression data.

^d^Total number of pathways with at least five genes.

^e^Number of human genes with protein sequences available for constructing the BTP table.
