# Supplementary material for: A search engine to identify pathway genes from expression data on multiple organisms
Source: BMC Syst Biol. 2007 May 4;1:20. doi: 10.1186/1752-0509-1-20 (PMC1878502; doi:10.1186/1752-0509-1-20)
Supplement: Additional file 1 — Appendix. Discussion of related work and proof of correctness of order statistics implementation. [file 1752-0509-1-20-S1.pdf]

APPENDIX

Related Work

Many clustering strategies have been applied to the analysis of gene expression data, including hierarchical clustering [2], principal components analysis [48, 49], self-organizing maps [5], and multidimensional scaling [3]. Although these approaches have provided many insights, they cannot be applied to large databases because they are computationally intensive. Clustering approaches that partition the genes into mutually exclusive sets force genes to belong to a single group even though some genes may be coregulated with multiple pathways under different conditions. In addition, global clustering uses the entire set of conditions to compute gene similarity and may not detect coexpression patterns in a small fraction of the database. Prior work [40, 50-52] has demonstrated the value of combining datasets across organisms to infer gene function. For example, the methods of Snel *et al.* [52] and Stuart *et al.* [40] both compute gene similarity using all of the experiments in a single organism's database before combining across species. Conversely, the methods of Bergmann *et al*. [50] and Lee *et al*. [51] search for informative experiments but do not combine the results from different organisms.

Recommender systems provide a complementary approach to clustering for predicting gene function because they use information about known members to identify unknown members [53]. Such systems can select for microarray experiments that show tight coregulation of the query genes and ignore uninformative experiments that would otherwise add noise to the calculations. As a result, they may generate shorter hit lists with a higher percentage of query genes than global clustering approaches. Recommender systems can naturally handle multifunctional genes. Genes that are expressed at different times during development or in

different tissues may interact with multiple pathways. A clustering approach such as hierarchical clustering or multidimensional scaling would place such multifunctional genes into the strongest cluster, and interactions with other clusters would be lost.

Correctness of the order statistics formulation

Our method for constructing a new search ordering from *n* organisms uses a formula that computes the joint *P* value associated with *n* rank-ratios. We could not find a proof that the recursive definition reported by [40] equals the joint *P* value of *n* order statistics, so we include a proof of its correctness here. Theorem 1 gives a recursive formula that uses order statistics to combine P values from multiple significance tests. Corollary 1 applies this theorem to the special case where the corresponding random variables are uniformly distributed on $[0,1]$. Both formulas provide an alternative to the standard method of multiplying *P* values to combine results from multiple significance tests. Theorem 1 has the advantage of applicability to large classes of distributions for the random variables, but has the disadvantage, along with Corollary 1, of taking exponential time to compute. Corollary 1 is useful in the context of this paper, since there are only six random variables, representing the six species used in the expression database. However, this formula is impractical if there are many random variables. Corollary 2 gives an alternative formula with a computation time that is quadratic in the number of random variables.

Let $X = (X_1, X_2, ..., X_n)$ be a vector of independent random variables on the real interval $[a, \infty]$ with corresponding cumulative distribution functions $F_i(r) = P(X_i < r),$ probability density functions p_i, and order statistics $X_{(i)} = i$ th smallest component in X. Suppose that R is a monotonically increasing sequence $\{r_0 = a, r_1, ... r_n\}$ of real numbers. If V is a sequence or vector, then V_i, V_{-i}, and $V_{i,j}$ refer respectively to the ith component in V, to V with the ith

component removed, and to the vector $(V_i, V_{i+1}, ..., V_j)$. Denote by $P_X(R)$ the probability that $X_{(i)} < r_i$ for $1 \le i \le n$. The following theorem gives a recursive formula for computing $P_X(R)$.

Theorem 1: If *$X, R,$ and $P_X(R)$ are as above, then*

$$P_X(R) = \sum_{j=1}^{n} (F_1(r_j) - F_1(r_{j-1})) P_{X_{-1}}(R_{-j}).$$

Proof: The theorem follows directly from translating the formula into a statement involving sets. Define $S_R = \{X : X_{(1)} < r_1, ..., X_{(n)} < r_n\}$ for $1 \le j \le n$ as the set of *n*-dimensional vectors with components constrained to be smaller than the ordered values in *R* and let $T_R^j = \{X \vdots r_{j-1} \le X_1 < r_j, X_{-1} \in S_{R_{-j}}\}$. It is clear that $P_X(R) = P_p(S_R)$, where P_p is the probability measure generated on $[a, \infty]^n$ by the probability density functions $p_1, \cdots, p_n$. It also follows that $P_p(T_R^j) = (F_1(r_j) - F_1(r_{j-1})) P_{X_{-1}}(R_{-j})$, and that the sets $\{T_R^j\}_{j=1}^n$ are mutually disjoint. To prove the theorem, it then suffices to show that $S_R = \bigcup_{j=1}^{n} T_R^j$. The following lemma is instrumental to proving this equation.

Lemma: *The order statistic $X_{(i)} < r$ if and only if there are i components of X that are less than r.*

Proof: If $X_{(i)} < r,$ then $\{X_{(1)},..., X_{(i)}\}$ gives a corresponding set of i components of X that are less than $r.$ If $X_{(i)} \geq r,$ then there are at most $i-1$ values of X, corresponding to the set $\{X_{(1)},..., X_{(i-1)}\}$ of order statistics, that are less than $r.$

Proof that $S_R = \bigcup_{j=1}^{n} T_R^j$: Suppose that $X \in S_R.$ It is clear that $r_0 \leq X_1 \leq X_{(n)} < r_n,$ and so there is a $j \geq 1$ such that $r_{j-1} \leq X_1 < r_j.$ Based on the definition of S_R and on the lemma, there are i components of X that are less than r_i for each i with $1 \leq i \leq n.$ Table A1 uses this observation together with the lemma to show that $(X_{-1})_{(i)} < (R_{-j})_i$ for each i with $1 \leq i \leq n-1.$ This proves that $X_{-1} \in S_{R_{-j}}.$ If $X \in T_R^j$ for some j with $1 \leq j \leq n,$ then $r_{j-1} \leq X_1 < r_j$ and $(X_{-1})_{(i)} < (R_{-j})_i$ for each i with $1 \leq i \leq n-1.$ It then follows from the lemma that there are i components of X_{-1} that are less than $(R_{-j})_i$ for each i with $1 \leq i \leq n-1.$ Table A2 uses this fact together with the lemma to show that $X \in S_R.$ This completes the proof of the theorem.

Table A1

Value of i	***Necessary*** i ***components of*** $\{X_2,..., X_n\}$ ***that are less than*** $(R_{-j})_i$
$i \leq j-1$	Same as the i components of X that are less than $r_i,$ since $(R_{-j})_i = r_i \leq r_{j-1} \leq X_1.$
$i > j-1$	Same as the $i+1$ components of X that are less than $r_{i+1},$ excluding $X_1,$ since $(R_{-j})_i = r_{i+1}.$

Table A2

Value of i	***Necessary*** i ***components of*** $\{X_1, X_2,..., X_n\}$ ***that are less than*** r_i
$i \leq j-1$	Same as the i components of X_{-1} that are less than $(R_{-j})_i$, since $(R_{-j})_i = r_i.$
$i = j$	Same as the $j-1$ components of X_{-1} that are less than $(R_{-j})_{j-1} = r_{j-1} < r_i,$ with X_1

	added.
$i > j$	Same as the $i-1$ components of X_{-1} that are less than $(R_{-j})_{i-1} = r_i$, with X_1 added.

The following corollary applies Theorem 1 where the random variables are all uniformly distributed on $[0,1]$.

Corollary 1: *If* $X_1, X_2, ..., X_n$ *are uniformly distributed on* $[0,1]$, *then* $P_X(R) = \sum_{j=1}^{n} (r_j - r_{j-1}) P_{X_{-1}}(R_{-j})$.

Suppose that $f(n)$ is the time required to naively compute the formula given in Corollary 1. Based on this formula, $f(n)$ satisfies the recurrence $f(n) \geq nf(n-1)$. If $f(1) = c$, then a straightforward induction argument shows that $f(n) \geq cn!$, so that the time required for naive computation is exponential. Computing this formula "bottom up," starting with the *P* values for the single components and storing intermediate results, reduces the computation time somewhat, but still requires exponential computation time. Theorem 2 and Corollary 2 seek to find a more efficient recursive formula in the case where the random variables are uniformly distributed on $[0,1]$.

Where $X_1, X_2, ..., X_n$ are independent and uniformly distributed on $[0,1], P_X(R)$ can be expressed as the following multiple integral: $n!\int_0^{r_1}\int_{s_1}^{r_2}\cdots\int_{s_{n-2}}^{r_{n-1}}\int_{s_{n-1}}^{r_n} ds_n ds_{n-1}\cdots ds_1$. Define $Q_{0,i} = 1$ for $i \geq 0$ and let $Q_{n,i}(R) = \int_0^{r_1}\int_{s_1}^{r_2}\cdots\int_{s_{n-2}}^{r_{n-1}}\int_{s_{n-1}}^{r_n} s_n^i ds_n ds_{n-1}\cdots ds_1$ for $i \geq 0$ and $n \geq 1$. Let $p(i, j)$ denote the

number of permutations of j objects from i objects. The following formula follows from a straightforward induction argument.

Theorem 2: If $i \geq 0$ **and** $n \geq 1,$ **then** $Q_{n,i}(R) = \sum_{j=1}^{n} (-1)^{j+1} r_{n-j+1}^{i+j} Q_{n-j,0}(R_{1,n-j}) / p(i+j, j).$

Proof: By induction on $n.$ If $n = 1,$ then $Q_{n,i}(R_{1,1}) = \int_0^{r_1} s_1^i ds_1 = r_1^{i+1}/(i+1).$ For the induction step, assume that the theorem is true for $n = k.$ It then follows that

$$Q_{k+1,i}(R) = \int_0^{r_1}\int_{s_1}^{r_2}\cdots\int_{s_{k-1}}^{r_k}\int_{s_k}^{r_{k+1}} s_{k+1}^i ds_k ds_{k-1}\cdots ds_1 = \int_0^{r_1}\int_{s_1}^{r_2}\cdots\int_{s_{k-1}}^{r_k}(r_{k+1}^{i+1} - s_k^{i+1})/(i+1) ds_k ds_{k-1}\cdots ds_1$$
$$= (r_{k+1}^{i+1} Q_{k,0}(R_{1,k}) - Q_{k,i+1}(R_{1,k}))/(i+1)$$
$$= (r_{k+1}^{i+1} Q_{k,0}(R_{1,k}) - \sum_{j=1}^{k} (-1)^{j+1} r_{k-j+1}^{i+1+j} Q_{k-j,0}(R_{1,k-j}) / p(i+1+j, j))/(i+1)$$
$$= r_{k+1}^{i+1} Q_{k,0}(R_{1,k})/(i+1) + \sum_{l=2}^{k+1} (-1)^{l+1} r_{k-l+2}^{i+l} Q_{k-l+1,0}(R_{1,k-l+1}) / p(i+l, l)$$
$$= \sum_{l=1}^{k+1} (-1)^{l+1} r_{(k+1)-l+1}^{i+l} Q_{(k+1)-l,0}(R_{1,(k+1)-l}) / p(i+l, l),$$

where the substitution in the second to last step is $l = j+1.$ This proves that the theorem is true for $n = k+1,$ and hence completes the proof of the theorem.

To translate Theorem 2 into a formula for computing $P_X(R),$ note that $P_X(R) = n! Q_{n,0}(R).$ Corollary 2 now follows directly from this equation together with Theorem 2. The notation $c(n, j)$ refers to the number of combinations of j objects from n objects.

Corollary 2: If $n \geq 1,$ ***then*** $P_X(R) = \sum_{j=1}^{n} (-1)^{j+1} c(n, j) r_{n-j+1}^{j} P_{X_{1,n-j}}(R_{1,n-j}).$

A “bottom up” approach to computing $P_X(R)$ computes $P_{X_{1,1}}(R_{1,1}),$ uses this result to compute $P_{X_{1,2}}(R_{1,2})$ using Corollary 2, and in general uses $P_{X_{1,1}}(R_{1,1}), P_{X_{1,2}}(R_{1,2}),..., P_{X_{1,j-1}}(R_{1,j-1})$ to compute $P_{X_{1,j}}(R_{1,j})$ for $j = 2,...,n.$ Assuming that the calculation of each component of each sum requires constant time $c,$ the calculation of $P_X(R)$ requires $\sum_{j=1}^{n} jc = cn(n+1)/2$ time. Corollary 2 gives a quadratic time method for computing $P_X(R),$ which is a dramatic improvement over the exponential time method given by Corollary 1.
