# Supplementary material for: A search engine to identify pathway genes from expression data on multiple organisms
Source: BMC Syst Biol. 2007 May 4;1:20. doi: 10.1186/1752-0509-1-20 (PMC1878502; doi:10.1186/1752-0509-1-20)
Supplement: Additional file 10 — Table S5. Summary of significant cis-regulatory motifs identified in the Collagens and Calcium Channels search results. [file 1752-0509-1-20-S10.pdf]

Table S5. Summary of significant *cis*-regulatory motifs identified in the *Collagens* and *Calcium Channels* search results

Group[1]	Factor[2]	Matrix ID[3]	Involvement[4]	In Group[5]	Out Group[5,6]	Enrichment[7]	Random *P*-value[8]
Collagens	EGR1	V$EGR1_01	Yes [1]	5/23 **	X	4.32	$1.78e^{-4}$
Collagens	EGR	V$EGR_Q6	Yes [1]	9/23 *	X	2.46	$1.01e^{-3}$
Collagens	EGR1 or EGR	V$EGR1_01 or V$EGR_Q6	Yes [1]	10/23 **	X	3.51	-
Ca++	CAC-binding protein	V$CACBINDINGPROTEIN_Q6	No	14/25 **	14/19 ***	2.05	$5.34e^{-4}$
Ca++	ZIC2	V$ZIC2_01	Yes [2]	8/25 **	1/19	2.91	$3.11e^{-4}$
Ca++	MZF1	V$MZF1_01	No	17/25 *	7/25	1.64	$2.79e^{-3}$
Ca++	MAZR	V$MAZR_01	Yes [3]	11/25 *	9/19	2.14	$2.31e^{-3}$
Ca++	ZIC1	V$ZIC1_01	Yes [2]	13/25 *	1/19	1.91	$3.23e^{-3}$
Ca++	MAZ	V$MAZ_Q6	Yes [3]	13/25 *	10/19	1.86	$3.67e^{-3}$
Ca++	POU3F2	V$POU3F2_02	Yes [4]	6/25 *	1/19	3.19	$8.67e^{-4}$

1. Name of search result. *Collagens* refers to the GenMAPP *Collagens* pathway while *Ca++* refers to the GenMAPP *Calcium Channels* pathway.
2. Name of transcription factor.
3. TRANSFAC [5] ID of matrix that characterizes the given transcription factor.
4. Evidence implicating the transcription factor in the corresponding pathway.
5. Number of genes in the search result with hits to the matrix, along with its *P*-value range: '***' corresponds to P < 0.001, '**' to P < 0.005, '*' to P < 0.01.
6. The "Out Group" refers to the query genes that the MSGR did not rank in the top 25. The out group for the *Collagens* search result was too small for the binding site analysis.
7. Enrichment of search results for the matrix as compared to the genomic background distribution.
8. *P*-value obtained from comparing the number of genes in the search result to random collections of the same number of genes.

1. Alexander D, Judex M, Meyringer R, Weis-Klemm M, Gay S, et al. (2002) Transcription factor Egr-1 activates collagen expression in immortalized fibroblasts or fibrosarcoma cells. Biol Chem 383: 1845-1853.
2. Aruga J (2004) The role of Zic genes in neural development. Mol Cell Neurosci 26: 205-221.
3. Ugai H, Li HO, Komatsu M, Tsutsui H, Song J, et al. (2001) Interaction of Myc-associated zinc finger protein with DCC, the product of a tumor-suppressor gene, during the neural differentiation of P19 EC cells. Biochem Biophys Res Commun 286: 1087-1097.
4. Fujii H, Hamada H (1993) A CNS-specific POU transcription factor, Brn-2, is required for establishing mammalian neural cell lineages. Neuron 11: 1197-1206.
5. Wingender E (2004) TRANSFAC, TRANSPATH and CYTOMER as starting points for an ontology of regulatory networks. In Silico Biol 4: 55-61.
