# Supplementary material for: A search engine to identify pathway genes from expression data on multiple organisms
Source: BMC Syst Biol. 2007 May 4;1:20. doi: 10.1186/1752-0509-1-20 (PMC1878502; doi:10.1186/1752-0509-1-20)
Supplement: Additional file 11 — Table S6. Sequences of potential cis-regulatory binding sites of transcription factors identified in the Calcium Channels search results. [file 1752-0509-1-20-S11.pdf]

Table S6. Sequences of potential *cis*-regulatory binding sites of transcription factors identified in the *Calcium Channels* search results

Gene[1]	CAC-binding protein[6]	ZIC2[2,7]	MZF1[8]	MAZR[9]	ZIC1[10]	MAZ[11]	POU3F2[12]
CACNB2	-	-	-	-	-	-	GTATGTTAAT[3]
CACNA1D	GGGGGTGGG[3]	-	GGTGGGGA	GGGGGCGGGGCCT	GGGGTGGCC	GGGGAGGG	TTATCTTAAT
CACNA1A	-	-	GGTGGGGA	CCGGGGGGGGCGT	TGGGTGGAC	GGGGAGGG	-
RIMS2	-	-	-	-	-	-	TTATGTGAAT
MADD	GGGGGTGGG	-	GGTGGGGA	GGGGGTGGGGACA	-	-	-
CNTN1	-	-	GGTGGGGA	TGGGGCGGGGACC	-	-	-
GAD1	-	-	AGAGGGGA	GGGGGAGGGGAAA	GGGCTGGTC	GGGGAGGG	-
RYR2	GGGGGTGGG	CGGGTGGTC	AGCGGGGA	-	CGGGTGGTC	-	-
HIVEP1	GGGGCTGGG	TGGGTGGTG	GGGGGGGA	CGGGGGGGGGAGC	TGGGTGGTG	GGGGAGGG	-
MME	GAGGGTGGG	-	GGTGGGGA	-	GGGGTGGGC	GGGGAGGG	-
STN2	-	GGGGTGGTT	-	-	-	-	TTATGTCAAT
CHRND	GAGGGTGGG	TGGGTGGTG	GGTGGGGA	AGGGGTGGGGCCC	TGGGTGGTG	GGGGAGGG	-
TGFB1	GGGGCTGGG	TGGGTGGTG	-	-	TGGGTGGTG	GGGGAGGG	-
SPTBN1	-	-	CGTGGGGA	-	GGGTTGGTC	-	-
SPTAN1	GGGGCTGGG	-	-	-	-	GGGGAGGG	-
SHOC2	-	-	AGCGGGGA	-	-	GGGGAGGG	-
PTPRN2	-	-	AGTGGGGA	GGGGGCGGGGCCC	-	GGGGAGGG	-
KCNK3	GAGGGTGGG	TGGGTGGTG	GGTGGGGA	TGGGGAGGGGCTC	TGGGTGGTG	GGGGAGGG	-
SLC6A2	GGGGCTGGG	CGGGTGGTC	-	-	CGGGTGGTC	GGGGAGGG	-
PTPN4	-	-	AGGGGGGA	GGGGCGGGGGCCA	-	-	-
RASSF1	GGGGCTGGG	TGGGTGGTC	TGTGGGGA	GCGGGGGGGGCTC	TGGGTGGTC	-	-
TNNT2	GGGGGTGGG	-	AGCGGGGA	-	GGGGTGGGC	-	TTATGTCAAT
ITPKB	GGGGCTGGG	-	AGCGGGGA	-	-	GGGGAGGG	-
USP11	-	-	-	-	-	-	-
TTN	GAGGCTGGG	-	-	-	-	-	TGATGTTAAT
**Matrix[4]**	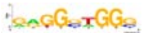	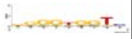	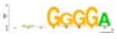	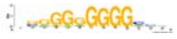	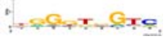	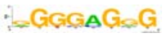	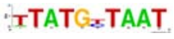
**Found[5]**	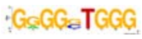	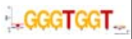	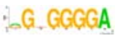	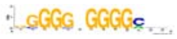	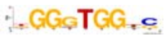	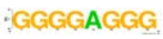	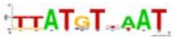

Significant neural-related transcription factor binding site hits found in the *Calcium Channels* search results.

1. Gene symbol.
2. Transcription factor name.
3. Sequence of the best binding site hit upstream of that gene for each binding matrix significant to the gene set. A dash indicates that no hit was found for that matrix upstream of that gene.
4. Sequence logo derived from the transcription factor's matrix. Each nucleotide of a sequence logo corresponds to one position of a binding site. The overall height of the stack at each position indicates the sequence conservation at that position, while the height of symbols within the stack indicates the relative frequency of each nucleic acid at that position.
5. Sequence logo derived from hits found in the gene set.
6. CBP corresponds to V$CACBINDINGPROTEIN_Q6.
7. ZIC2 corresponds to the TRANSFAC matrix V$ZIC2_01.
8. MZF1 corresponds to V$MZF1_01.
9. MAZR corresponds to V$MAZR_01.
10. ZIC1 corresponds to V$ZIC1_01.
11. MAZ corresponds to V$MAZ_Q6.
12. POU3F2 corresponds to V$POU3F2_02.
