# Supplementary material for: A search engine to identify pathway genes from expression data on multiple organisms
Source: BMC Syst Biol. 2007 May 4;1:20. doi: 10.1186/1752-0509-1-20 (PMC1878502; doi:10.1186/1752-0509-1-20)
Supplement: Additional file 12 — Table S7. Sequences of potential cis-regulatory binding sites identified in the Collagens search results. [file 1752-0509-1-20-S12.pdf]

Table S7. Sequences of potential *cis*-regulatory binding sites identified in the *Collagens* search results

Gene[1]	EGR1[2,6]	EGR[7]
COL4A2	**GTGCGCGGGCGG**[3]	GAGGGGGCAGC
NID	-	GTGGGAGCTGG
ABLIM1	-	-
HSPG2	**CTGCGGGGGCGG**	**GCGGGGGCGGG**
SPTAN1	-	**GTGGGGGCTGG**
SPARC	-	-
LAMA2	-	GTGGGGGAGAA
LAMB1	**ATGCGTGGGGGC**	**GTGGGGGCGTC**
COL4A5	CTGCGAGGGAGT	-
ATXN2	-	**GTGGGGGCAAA**
LAMC1	CGGCGGGGGCGG	**GCGGGGGCGGG**
STXBP1	GCGCGGGGGCGG	**GCGGGGGCGGG**
CALU	**TGGCGTGGGCGC**	**GTGGGCGCGGC**
HDLBP	TTGGGTGGGCCT	-
CRYAB	GTGCGGGGGAGG	**GTGGGGGCGGG**
COL15A1	-	GCGGGCGCGGG
ATP6AP1	TCGGGTGGGCGT	GCGGGGGCAAC
PLOD3	GTGAGTGGGAGG	-
CNTN1	-	-
ARK5	-	-
FLNA	**GTGCGTGGGGGG**	**GTGGGGGCAAC**
AASS	-	-
SPTBN1	-	-
**Matrix[4]**	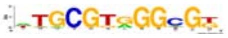	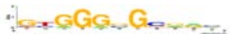
**Found[5]**	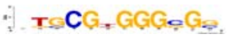	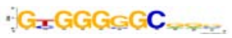

Significant neural-related transcription factor binding site hits found in the *Collagens* search results. Sequences in bold represent hits identified by the more stringent analysis. Non-bold hits correspond to sequences that have binding matrix scores greater than the genome-wide mean score for that binding matrix.

1. Gene symbol.
2. Transcription factor name.
3. Sequence of the best binding site hit upstream of that gene for each binding matrix significant to the gene set. A dash indicates that no hit was found for that matrix upstream of that gene.
4. Sequence logo derived from the transcription factor's matrix. Each nucleotide of a sequence logo corresponds to one position of a binding site. The overall height of the stack at each position indicates the sequence conservation at that position, while the height of symbols within the stack indicates the relative frequency of each nucleic acid at that position. Only the bold (stringent) hits were used to create the sequence logos.
5. Sequence logo derived from hits found in the gene set.
6. EGR1 corresponds to the TRANSFAC matrix V$EGR1_01.
7. EGR corresponds to V$EGR_Q6.
