# Supplementary material for: A search engine to identify pathway genes from expression data on multiple organisms
Source: BMC Syst Biol. 2007 May 4;1:20. doi: 10.1186/1752-0509-1-20 (PMC1878502; doi:10.1186/1752-0509-1-20)
Supplement: Additional file 13 — Table S8. Conservation of predicted binding sites in the Collagens search result. [file 1752-0509-1-20-S13.pdf]

Table S8. Conservation of predicted binding sites in the *Collagens* search result

**Gene**	CRYAB
**Query?**	No
**Conserved?**	Yes
**Location**	chr11:111288656-111288666
**Score**	10.076
**Alignment:**	
Human	CCCGCCCCCAC
Chimp	CCCGCCCCCAC
Rhesus	CCCGCCCCCAC
Mouse	CCCGCCCCCA-
Rat	CCCGCCCCCA-
Rabbit	CCCGCCCCCAC
Cow	CCCGCCCCCAC
Dog	CCCGCCCCCAC
Armadillo	CCCGCCCCCAC
Elephant	CCCGCCCCCAC
Tenrec	CCCGCCCCCAC
Opossum	CCCGCCCCAGC

**Gene**	COL4A2
**Query?**	Yes
**Conserved?**	Yes
**Location**	chr13:109757560-109757571
**Score**	8.125
**Alignment:**	
Human	GTGCGCGGGCGG
Chimp	GTGCGCGGGCGG
Rhesus	GTGCGCGGGCGG
Mouse	GTGCGCGGGCGG
Rat	GTGCGCGGGCGG
Rabbit	GTGCGCGGGCGG
Armadillo	GTGCGCGGGCGG
Elephant	GTGCGCGGGCGG
Opossum	GTGCGCGGGCGG

**Gene**	SPTAN1
**Query?**	No
**Conserved?**	Yes
**Location**	chr9:128393814-128393824
**Score**	6.662
**Alignment:**	
Human	CCAGCCCCCAC
Chimp	CCAGCCCCCAC
Rhesus	CCAGCCCCCAC
Rat	CCAGCCCCCAC

Mouse	CCATCCCCCAC
Rabbit	ACGCCCCC---
Cow	TCA----CCAG
Dog	CCAGCCCCCAG
Armadillo	CCAGTCCTCAT
Elephant	CCAGCCCCCAT
Tenrec	CCAGGCCTCAT

**Gene**	STXBP1
**Query?**	No
**Conserved?**	Yes
**Location**	chr9:127454033-127454043
**Score**	5.667
**Alignment:**	
Human	CCCGCCC-------------CCGC
Opossum	CCCACCCCTCCCAACGATATCCGA
Tenrec	---------------------CGC
Elephant	-CCGCCCCC-----------CCGC
Dog	CCCGCCCGC---------GCGCGC
Cow	----------------------GC
Mouse	CCCGCCCCT----------CCCGC
Rat	CCCGCCTCT-----------CCGC
Chimp	CCCGCCC-------------CCGC

**Gene**	HSPG2
**Query?**	Yes
**Conserved?**	Yes
**Location**	chr1:22009148-22009160
**Score**	5.005
**Alignment:**	
Human	CCCGCCCCCGCAG
Rhesus	CCCGCCCCCGCGG
Mouse	CCCGCCCCCGGGT
Rat	CCCGCCCCCGGGT
Cow	CCCCCC-------
Dog	CCCGCC-------
Armadillo	TCCGCCCCCCTAG

**Gene**	LAMC1
**Query?**	Yes
**Conserved?**	Yes
**Location**	chr1:179724028-179724038
**Score**	4.745
**Alignment:**	
Human	C---CCGCCCCCGC
Chimp	C---CCGCCCCCGC
Rhesus	----CCGCCCCCGC

Mouse	----CCGCCCCCGC
Cow	----CTGC------
Armadillo	CCCGCCGCCTCCGC
Elephant	----CCGCCGCCGC
Tenrec	----CCGCCCCCGC

**Gene**	LAMB1
**Query?**	Yes
**Conserved?**	Yes
**Location**	chr7:107237824-107237838
**Score**	4.555
**Alignment:**	
Human	ATGCGTGGGGGC--GTC
Chimp	ATGCGTGGGGAC--GTC
Rhesus	ATGCATGGGGGC--GTC
Rat	ATGCGTTGGGGCTGGTC
Mouse	ATGCGTCGGGGCTGGTC
Rabbit	ATGCGTTGGGGCTGGTC
Cow	AGGCGTTCGGGCGGGTC
Armadillo	ACCCTTTCGGGTTGGTG
Elephant	ATGTGGCGGGGCTGGTC
Tenrec	ATGAGTCCCGGCTGGCC
Opossum	ATGAGTACTGACTGCTC

**Gene**	ATXN2
**Query?**	No
**Conserved?**	Yes
**Location**	chr12:110500376-110500386
**Score**	2.334
**Alignment:**	
Human	TT-------TG-CCCCCAC
Chimp	TT-------TG-CCCCCAC
Rhesus	TT-------TG-CCCCCAC
Mouse	ATTTCACCCCA-CCCCCAC
Rabbit	GT-------CG-CCCCCAC
Cow	TT-------TG-GCCCCAC
Dog	TT-------TA-CCCCCAC
Armadillo	AT----------CCCCCAC
Tenrec	CT-------GGTCCCCCAC

**Gene**	FLNA
**Query?**	No
**Conserved?**	Yes
**Location**	chrX:153123969-153123980
**Score**	1.574
**Alignment:**	
Human	GTGCGTGGGGGG

Chimp	GTGCGTGGGGGG
Rhesus	GTGCGTGGGGGG
Mouse	G-----GAGTGG
Rat	G-----GAGGGG
Rabbit	G-----GAGGGG
Cow	G-----------
Dog	G-----------

Gene CALU
Query? No
Conserved? Yes
Location chr7:127973080-127973094
Score 1.382
Alignment:

Human	GCCGCGCCCA---CGCCA
Chimp	GCCGCGCCCA---CGCCA
Rhesus	GCCGCGCCCA---CGCCA
Rat	GCCACGGTCAGTGAGCCG
Mouse	GCCAAGGTCAGCGAGCCG
Rabbit	GCCGCGCCCA-CAGGCCG
Cow	GCCGCGCCCA-CACGGCA
Dog	GCCGCGCCCA-CACGCCG
Tenrec	GCCGCGCCCA-C-TGCCG
Elephant	ACCGCGCCCA-CACGCCA
Opossum	ACTTCGTCCC---CCACC

Gene HSPG2
Query? Yes
Conserved? No
Location chr1:22009205-22009216
Score -1.875
Alignment:

Human	CCGCCCACGGA-----------------------------C
Rhesus	CCGCCCCCGGA-----------------------------C
Mouse	CCACCTCCAAACATTTAGCCACACCCCACAAGACCAGGTTC
Rat	CCACCTTCAAACATCTAGCCACGCCCTACAAGACCAGGTTC
Cow	CCACCCCCTAG-ACGAGGCCCCGCCCCGGA----------C

Gene FLNA
Query? No
Conserved? No
Location chrX:153124292-153124302
Score -7.593
Alignment:

Human	GGTG-GGG---GCAA
Chimp	GGTG-GGG---GCAA
Rhesus	GGTG-GGG---GCAA

Mouse	GATGAGAG---ATAA
Cow	AGTG-GGAAGTGCAA
Dog	GTGG-GGTCGCGCGG
Armadillo	GGTA-GAG---AGTG
