# Supplementary material for: A search engine to identify pathway genes from expression data on multiple organisms
Source: BMC Syst Biol. 2007 May 4;1:20. doi: 10.1186/1752-0509-1-20 (PMC1878502; doi:10.1186/1752-0509-1-20)
Supplement: Additional file 2 — Figure S1. Precision-recall results of the MSGR run on the GenMAPP test pathways. Plotted are the same results as shown in Figure 5A of the text, but the precision estimates have here been augmented to include standard error bars to show the variability of the MSGR's performance across the pathways. [file 1752-0509-1-20-S2.pdf]

Because the number of pathways and the number of genes in each pathway are small, we expect variability in our precision estimates. To determine if the precision of the multiple-species search results is significantly higher than that of the single-species searches given this expected variability, we calculated the average precision and average recall across five-fold cross-validation as described in the text. The average and standard deviation of the average precision across the pathways for each average recall level was then computed (see Figures S1A-C).

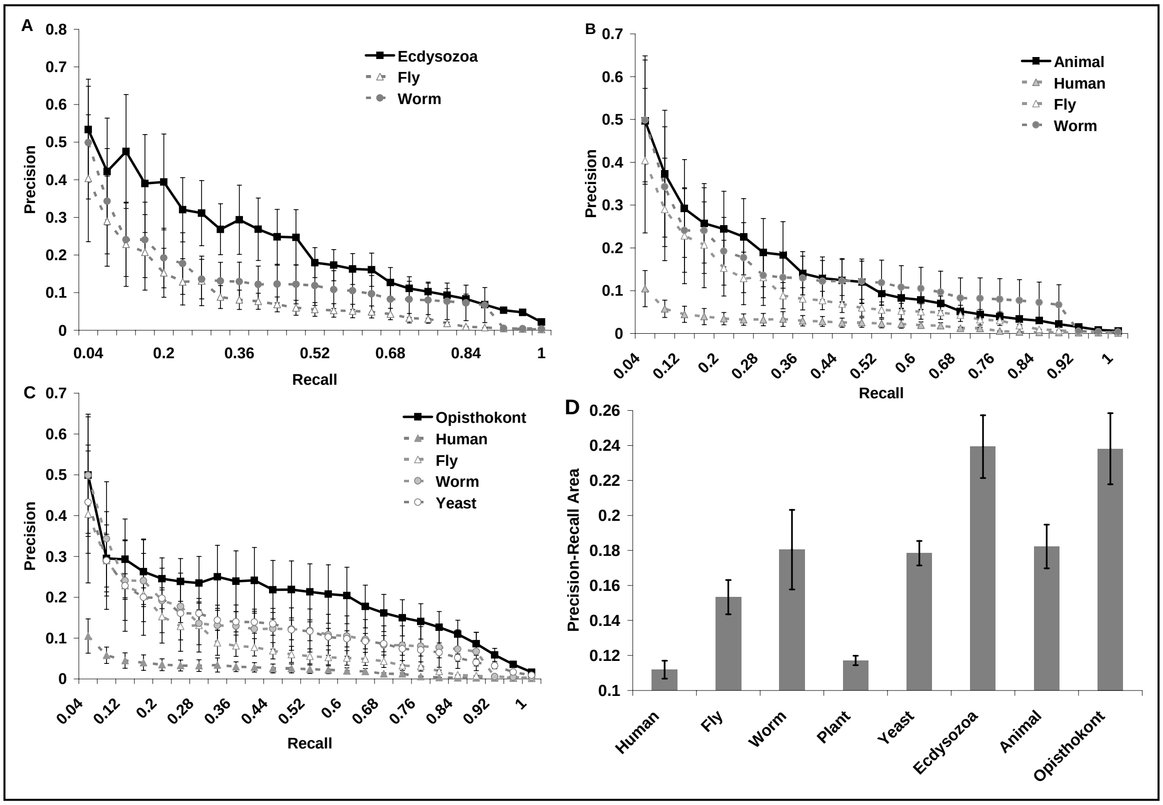

Figure S1. Precision-recall results of the MSGR run on the GenMAPP test pathways. Plotted are the same results as shown in Figure 5A of the text, but the precision estimates have here been augmented to include standard error bars to show the variability of the MSGR's performance across the pathways. **A-C.** The average precision achieved during cross-validation across the pathways is plotted against the average recall. Error bars represent two standard errors. **D.** The area under the precision-recall curve calculated for each pathway. Each bar's height indicates the average area for a specific search node. The standard error of the area across the pathways was calculated; error bars indicate two standard errors.

The multiple-species search results had higher average precision levels than single-species results across the majority of average recall levels, even though these levels are not separated by more than two standard deviations at any given recall level. To assess whether this observation is significant, the area under the precision-recall curve for each individual GenMAPP pathway was calculated. The area ranges from 0 to 1, with 0 indicating poor performance and 1 perfect performance. We plotted the mean and

standard error of the areas across the pathways in Figure S1D. The areas under the curve for both Ecdysozoa and Opisthokont were separated by over two standard deviations from the best single-species result (in this case Worm and Yeast). Predictions at the Animal node were not significantly more precise than predictions at the Worm node. Note that they were also not significantly better than predictions at the Yeast node, but the performance of the Yeast search is irrelevant with respect to the Animal search. These findings suggest that the overall performance of the MSGR can significantly improve over the single-species searches when all levels of recall are taken into consideration.
