# Supplementary material for: A search engine to identify pathway genes from expression data on multiple organisms
Source: BMC Syst Biol. 2007 May 4;1:20. doi: 10.1186/1752-0509-1-20 (PMC1878502; doi:10.1186/1752-0509-1-20)
Supplement: Additional file 3 — Figure S2. Robustness of the Collagens search result to various fractions of held-out data. [file 1752-0509-1-20-S3.pdf]

To test the sensitivity of the results returned by the MSGR to perturbations in the input data for the *Collagens* search result, an increasing amount of random microarray hybridizations for each organism (5% to 90%) was removed. Specific microarray hybridizations were withheld randomly and independently. Using the smaller datasets, we recorded the top 50 genes returned by the MSGR when the *Collagens* query was used to search the Human, Fly, Worm, Ecdysozoa, and Animal nodes.

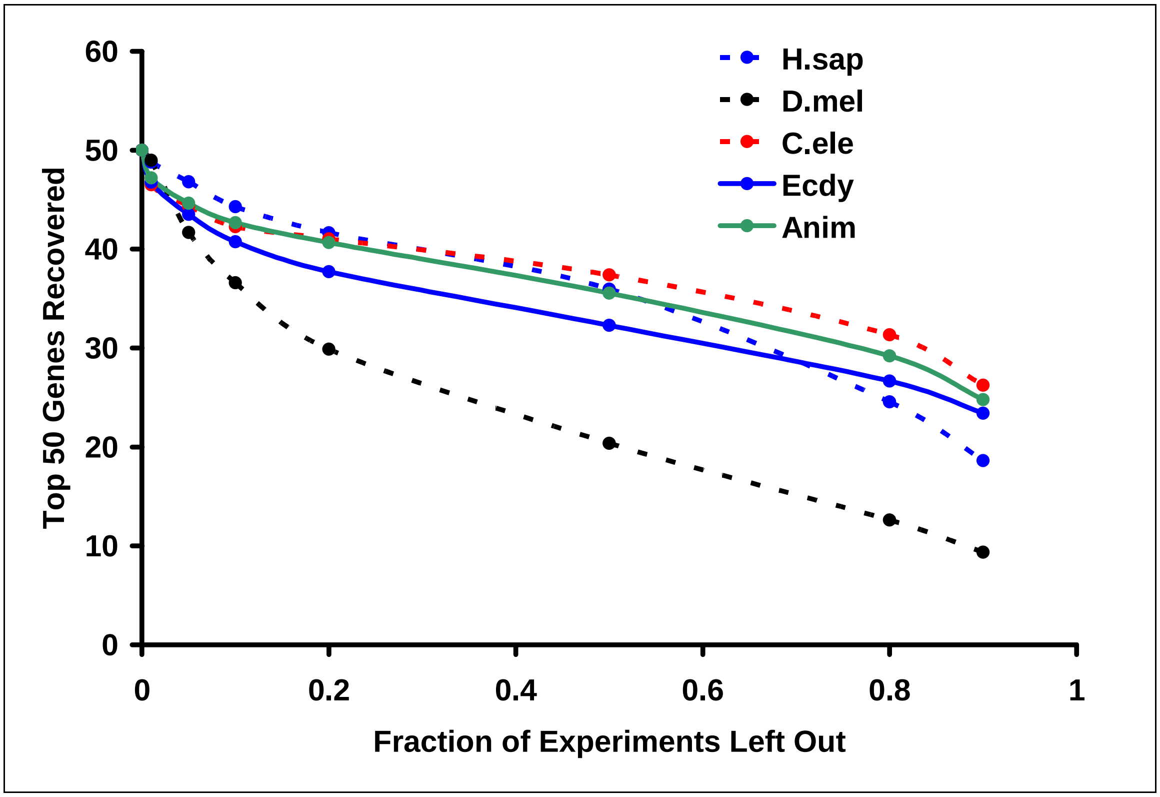

Figure S2. Robustness of the *Collagens* search result to various fractions of held-out data. Plotted is the number of genes recovered from the original top 50 *Collagens* search result (*y*-axis) for various fractions of held-out data (*x*-axis).

The search engine provided robust results even when large amounts of data was withheld. For example, even when 90% of the data was withheld, the search engine found 20-60% of the top 50 hits. The small slope in the 20-80% range indicates that the method is robust to a wide range in the amount of missing data. The search results obtained at multiple-species search nodes, Ecdysozoa and Animal, appear to be as robust as the single-species results, indicating that perturbations in the data do not propagate errors up the phylogenetic merge tree.
