# Supplementary material for: A search engine to identify pathway genes from expression data on multiple organisms
Source: BMC Syst Biol. 2007 May 4;1:20. doi: 10.1186/1752-0509-1-20 (PMC1878502; doi:10.1186/1752-0509-1-20)
Supplement: Additional file 4 — Figure S3. Performance of single-species search using query sets restricted to conserved genes. [file 1752-0509-1-20-S4.pdf]

To test whether the performance increase observed for the MSGR simply reflected restriction of the query genes conserved in human, worm, and fly, the cross-validation analysis was repeated for the human search described in the text with one modification: we restricted the query genes to those having predicted orthologs across all species under the Ecdysozoa PMT node. The precision levels achieved by these human searches for every GenMAPP pathway are shown in Figure S3.

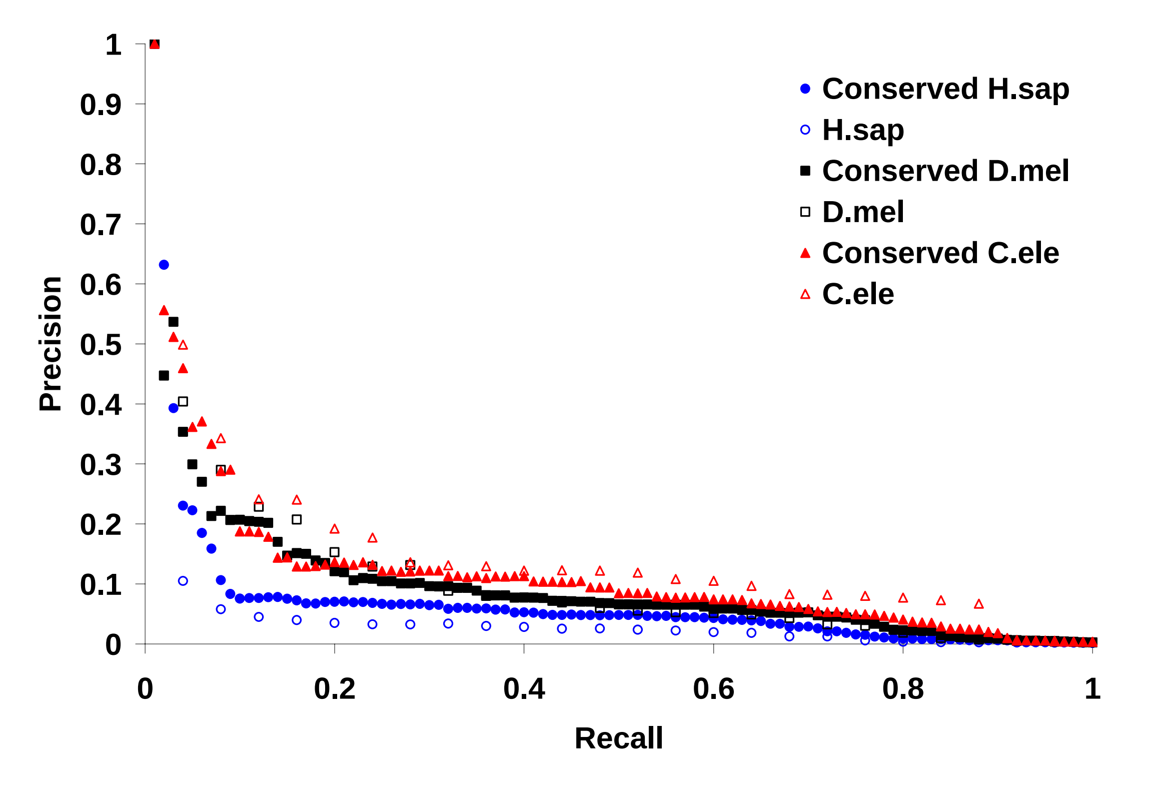

Figure S3. Performance of single-species search using query sets restricted to conserved genes. Average precision level across all of the GenMAPP pathways (*y*-axis) for each level of average recall (*x*-axis) measured during five fold cross-validation described in the text. Open circles correspond to the results obtained using all genes in the pathway; closed circles correspond to results obtained using queries containing only human genes with a predicted orthologs across all species under the Ecdysozoa PMT node.

The MSGR search results for queries of conserved genes only were comparabe to those obtained with the full set of query genes. Thus, restricting the search to conserved members of a pathway does not, in general, improve the performance of the search.
