# Supplementary material for: A search engine to identify pathway genes from expression data on multiple organisms
Source: BMC Syst Biol. 2007 May 4;1:20. doi: 10.1186/1752-0509-1-20 (PMC1878502; doi:10.1186/1752-0509-1-20)
Supplement: Additional file 5 — Figure S4. Performance as a function of orthology prediction error. [file 1752-0509-1-20-S5.pdf]

To quantify the performance of the MSGR as a function of the error in the predicted orthologs, a random fraction of the orthology was permuted and the MSGR was rerun on the 39 GenMAPP pathways. The average precision for every level of average recall at the Ecdysozoa node was then recorded (see Figure S4).

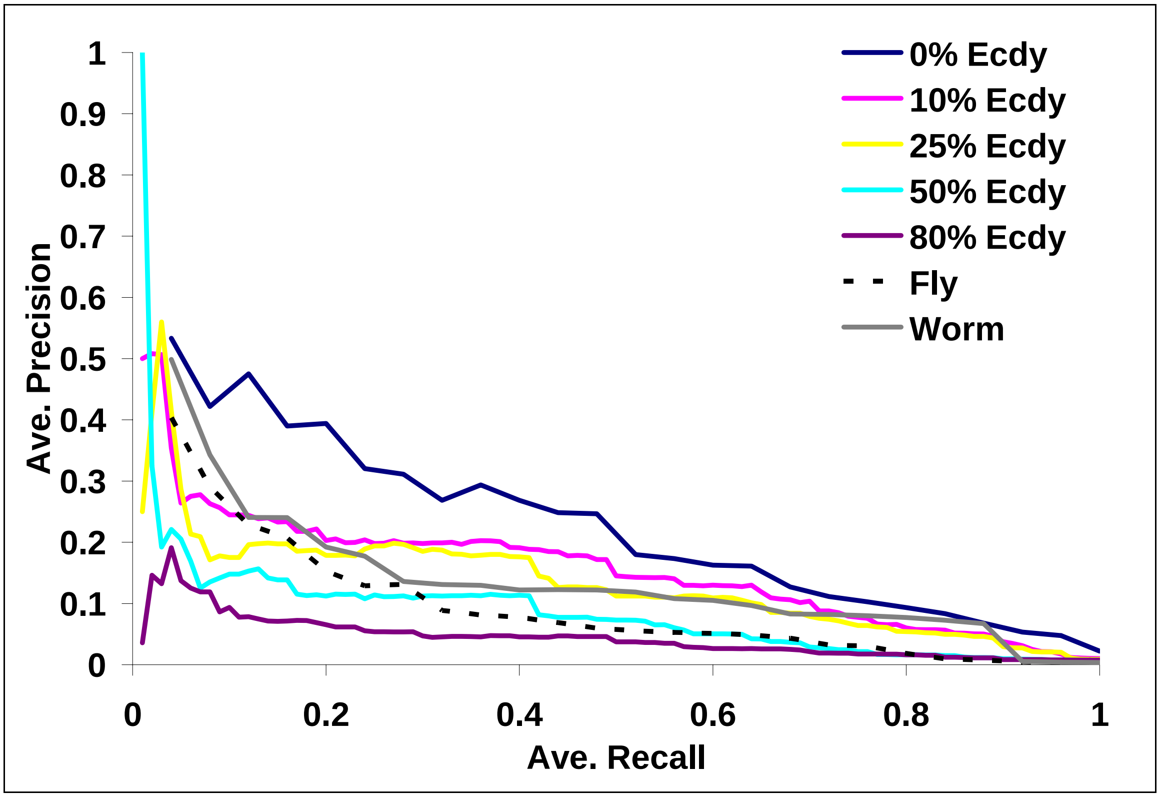

Figure S4. Performance as a function of orthology prediction error. Plotted is the average precision for each average recall level across the GenMAPP test pathways. Each line corresponds to a different percent of permuted orthology. The blue line shows the search results using the orthologs as specified by the best target proteins used in the text. The other lines correspond to permuting 10% (pink), 25% (yellow), 50% (light blue), and 80% (purple) of the worm and fly best target proteins for each of the 39 human queries. The accuracy of the search results obtained at the Fly (black dashes) and Worm (gray line) search nodes have been copied from Figure 5A and redrawn here.

As expected, the accuracy of the search did depend on the accuracy of the orthology prediction. Almost all of the accuracy is lost when 50-80% of the orthology assignments have been randomized. The accuracy was also significantly diminished when 10-25% of the orthology assignments were permuted. The Ecdysozoa search results were still comparable to those of the Worm and Fly searches at this range of permutation. These observations suggest that at least 75% of the orthology assignments, as predicted by BLAST, should be accurate to achieve the level of performance seen in the single-species searches. If the orthology prediction is less accurate, combining the searches across species appears to add enough noise to decrease the performance.
