# Supplementary material for: A search engine to identify pathway genes from expression data on multiple organisms
Source: BMC Syst Biol. 2007 May 4;1:20. doi: 10.1186/1752-0509-1-20 (PMC1878502; doi:10.1186/1752-0509-1-20)
Supplement: Additional file 14 — Table S9. Pathways for which accurate predictions with the MSGR could be made. [file 1752-0509-1-20-S14.pdf]

Table S9. Pathways for which accurate predictions with the MSGR could be made.

Pathway	Node with best precision at 50% recall
Kegg_Citrate_cycle_TCA_cycle	Cellular
GenMapp_TCA_Cycle	Cellular
Kegg_ATP_Synthesis	Eukaryote
GoList_collagen	Ecdysozoa
[illegible] achinery	Eukaryote
Biocarta_Electron_Transport_Reaction_in_Mitochondria	Cellular
Kegg_Glycolysis_or_Gluconeogenesis	Opisthokont
GenMapp_Peptide_GPCRs	Ecdysozoa
Kegg_Cysteine_metabolism	Eukaryote
GoList_electron_carrier_activity	Ecdysozoa
Biocarta_NFAT_and_Hypertrophy_of_the_heart_Transcription_in_the_broken_heart	Ecdysozoa
Kegg_Carbon_fixation	Ecdysozoa
Kegg_Pentose_phosphate	Cellular
GenMapp_Cell_cycle	Ecdysozoa
GoList_clathrin_coat	Opisthokont
Kegg_Cell_cycle	Ecdysozoa
GoList_ion_transporter_activity	Cellular
GoList_ATP-binding_cassette_ABC_transporter_activity	Cellular
Biocarta_Y_branching_of_actin_filaments	Opisthokont
Kegg_Purine_metabolism	Cellular
Kegg_Propanoate_metabolism	Opisthokont
GenMapp_Calcium_Channels	Worm
GenMapp_Cytoplasmic_Ribosomal_Proteins	Cellular
Kegg_Ribosome	Cellular
Kegg_Proteasome	Animal
GenMapp_Electron_Transport_Chain	Opisthokont
Kegg_Oxidative_phosphorylation	Opisthokont
GenMapp_Cytoplasmic_tRNA_Synthetases	Cellular
Kegg_Pyruvate_metabolism	Ecdysozoa
Biocarta_Glycolysis	Opisthokont
GoList_structural_constituent_of_ribosome	Opisthokont
GenMapp_Proteasome_Degradation	Opisthokont
Kegg_Aminoacyl-tRNA_biosynthesis	Cellular
GenMapp_Glycolysis_and_Gluconeogenesis	Eukaryote
GoList_heat_shock_protein_activity	Yeast
Biocarta_Regulation_of_PGC-1a	Yeast
GoList_nucleosome	Yeast
GenMapp_Fatty_Acid_Degradation	Worm
Kegg_Fatty_acid_biosynthesis_path_2	Fly
Kegg_Sterol_biosynthesis	Yeast
